# Lagrangian gradient regression for the detection of coherent structures from sparse trajectory data

**DOI:** 10.1098/rsos.240586

**Published:** 2024-10-30

**Authors:** Tanner D. Harms, Steven L. Brunton, Beverley J. McKeon

**Affiliations:** ^1^ Graduate Aerospace Laboratories, California Institute of Technology, Pasadena, CA 91106, USA; ^2^ Department of Mechanical Engineering, University of Washington, Seattle, WA 98195, USA; ^3^ Stanford University, Center for Turbulence Research, Palo Alto, CA, USA

**Keywords:** Lagrangian coherent structures, environmental flows, Lagrangian particle tracking, sparse gradient estimation, fluid dynamics, dynamical systems

## Abstract

Complex flows are often characterized using the theory of Lagrangian coherent structures (LCS), which leverages the motion of flow-embedded tracers to highlight features of interest. LCS are commonly employed to study fluid mechanical systems where flow tracers are readily observed, but they are broadly applicable to dynamical systems in general. A prevailing class of LCS analyses depends on reliable computation of flow gradients. The finite-time Lyapunov exponent (FTLE), for example, is derived from the Jacobian of the flow map, and the Lagrangian-averaged vorticity deviation (LAVD) relies on velocity gradients. Observational tracer data, however, are typically sparse (e.g. drifters in the ocean), making accurate computation of gradients difficult. While a variety of methods have been developed to address tracer sparsity, they do not provide the same information about the flow as gradient-based approaches. This work proposes a purely Lagrangian method, based on the data-driven machinery of regression, for computing instantaneous and finite-time flow gradients from sparse trajectories. The tool is demonstrated on a common analytical benchmark to provide intuition and demonstrate performance. The method is seen to effectively estimate gradients using data with sparsity representative of observable systems.

## 1. Introduction

To study a dynamical system by observing the trajectories of embedded tracers is to consider it in the Lagrangian frame. Many modern applications naturally admit a Lagrangian description. Ocean drifters monitored by global positioning system (GPS) [1], Arctic ice floes [2] and plastic pollution [3] are all examples of geophysical systems where the objects of interest serve as tracers in the flow and can be used to infer underlying oceanic dynamics. Particle flows are also common in the fluids laboratory where quantitative flow-field measurements are taken using passive tracers with techniques like particle image velocimetry (PIV) [4] or particle tracking velocimetry (PTV) [5,6]. Lagrangian methods are also applicable beyond the scope of fluid flows, e.g. for research into the movement of cells in a developing embryo [7] and protein folding [8].

Lagrangian particles represent observable instances of some underlying dynamical system that governs their behaviour. Coherent patterns exist in these dynamics, and identifying them is a critical task not only for scientific understanding but also for engineering applications. To this end, much work has been devoted to extracting and characterizing coherent structures from high-dimensional data [9]. The theory of Lagrangian coherent structures (LCS) uses tracer trajectories to characterize coherence [10]. Since the inception of the field, several excellent reviews and textbooks have been written, including Shadden [11], Haller [12], Allshouse & Peacock [13], Hadjighasem *et al*. [14] and Haller [15], and the tools of the field have been broadly applied. For example, LCS are often employed in the study of geophysical and atmospheric flows [16–21]. They have also been useful in characterizing structures in turbulent and unsteady flows of aerodynamic interest [22–25] and in biomedical flows [26–32]. Moreover, since LCS theory applies to general dynamical systems, it has also been applied in studies beyond the scope of fluid dynamics [7,8,33].

The properties of LCS make them particularly useful for the study of unsteady, transport-dominated flows. One of the principal characteristics of LCS is objectivity [12,34,35], which guarantees that identified features are invariant to translation and rotation. It allows for reliable results in systems where the relationship between the observer and the flow is ambiguous or dynamic. Another defining feature of LCS is the finite-time domain over which it is computed. Owing to their dependence on tracer trajectories, Lagrangian metrics must be computed over some time $t\in [t_{0},t_{0}+\Delta t],$ where 0<|Δt|<∞ . This distinguishes Lagrangian metrics from other common metrics such as the Q -criterion [36], $\lambda_{2}$ -criterion [37] and other field-based metrics [38,39], which do not give insight into the finite-time behaviour of unsteady flows.

Researchers have separated LCS detection methods into dense and sparse approaches [13], where dense approaches are further categorized as either geometric [12,40] or probabilistic [41,42]. The geometric approach bases its analyses on flow gradients and, therefore, detects features with specified dynamical interpretations. For instance, typical geometric analyses such as finite-time Lyapunov exponent (FTLE) [40] and Lagrangian-averaged vorticity deviation (LAVD) [43] physically represent the linear deformation rate of the flow and the amount of flow rotation over some analysis duration, respectively. The probabilistic approach to LCS is grounded on the Perron–Frobenius (or transfer) operator [44] and defines structures as regions exhibiting coherent behaviour with high probability. While probabilistic measures will often highlight the same spatial domain as corresponding geometric analyses (e.g. finite-time entropy [42] is a probabilistic proxy for FTLE), the field values are probabilistic in nature and are therefore not physically interpretable in the same way as geometric analyses. Both geometric and probabilistic methods are deterministic in the sense that they provide repeatable results when applied twice on the same flow.

Dense approaches can be difficult to use with practical datasets, however, since they require particle densities greater than that typically achievable in the field or in the lab. Many variations of sparse approaches for LCS detection have been proposed in the literature, including methods based on spectral clustering [45], fuzzy C-means [46], graph colouring [8,47], finite-element approximation of the dynamic Laplacian [48,49], network analysis [50] and density-based spatial clustering of applications with noise [7], among others. These methods are able to provide meaningful results with far fewer particles than dense alternatives, but they often lack determinism and do not typically provide quantifiable information on the flow kinematics. Often, users of sparse clustering methods must specify the number of structures to identify *a priori*, implying a prior understanding of the flow. So, while LCS may be identified with limited data, their interpretation requires knowledge of the flow. It remains a principal direction of LCS research to identify fast methods on sparse data that yield results with physical units and intuition.

Significant progress has been made towards structure detection on practical datasets. To overcome the dependency on a structured array of particles, Lekien & Ross [51] implemented least-squares regression to compute the Jacobian on an unstructured mesh of particles. Brunton & Rowley [52] developed an algorithm utilizing flow map composition to speed up the calculations of FTLE fields, which decreased FTLE computation time for successive frames by reusing prior computations. Raben *et al*. [53] built upon these advances to develop strategies for computing FTLE directly from experimental particle trajectories, circumventing velocity-field computations and improving computational efficiency. Recently, Mowlavi *et al.* [7] proposed a noise-robust method that augmented the regression approach of Lekien & Ross [51] by increasing the number of particle connections within the regression neighbourhood and by adding Tikhonov regularization. Another approach to the sparse identification of physics-based LCS exists in the recent work by Haller *et al*. (2021–2023) [54–57], where the trajectory stretching exponent (TSE) and trajectory rotation angle (TRA) have been developed as quasi-objective metrics that approximate traditional metrics like FTLE and polar rotation angle (PRA) and can be computed from single-particle trajectories [54,55]. The theoretical development of the deformation velocity by Kaszas *et al*. [56] has enabled the TSE and TRA to be computed objectively when using reference velocity information [57]. These methods were shown to be effective at visualizing LCS on sparse datasets, but they remain only an approximation of the actual FTLE, PRA and LAVD.

This work proposes a method capable of computing flow gradients—both velocity gradients and flow-map Jacobians—directly from sparse trajectory data. Unlike the work of Lekien and Ross [51] and Mowlavi *et al*. [7], the proposed approach performs regression over short durations (where the deformation can still be linearly approximated) and stitches them together using composition. Essentially, where traditional gradient-based approaches linearize in space, the proposed method linearizes in time and can, therefore, achieve significantly higher-accuracy gradients from spatially sparse data. The specific contributions of this work are here enumerated:

1. Lagrangian gradient regression (LGR) provides a novel method for approximating the velocity gradient tensor from trajectory data in a way that is free of numerical differentiation and interpolation. Therefore, it can be formatted so that it is robust to noise and is algorithmically space efficient.
2. By leveraging trajectory resolution in time, LGR enables accurate approximation of the flow-map Jacobian over arbitrary time intervals from sparse data, significantly outperforming other methods of sparse Jacobian approximation.
3. When clean velocity gradients are available, LGR enables objective single-trajectory computation of Jacobians.
4. By approximating velocity gradients, LGR provides a natural extension to the computation of rotational measures, such as LAVD, directly from trajectories.

The structure of the paper is as follows: §2 provides the prerequisite background and theory of geometric LCS and addresses common computational methods for computing geometric quantities. Section 3 considers the computation of geometric LCS quantities as the observation duration approaches zero. This will lay the groundwork for the finite-time methods which come in §§4 and 5, where methods for computing hyperbolic (stretch-based) and elliptic (rotation-based) structures will be developed for sparse trajectories. Finally, §6 demonstrates the methods on random fields of particles to gauge the usefulness of the developed tools for experimental and field data.

## 2. Preliminary theory of geometric LCS

Geometric LCS theory is an approach to characterize coherent structures according to the continuum mechanical properties of a flow [12]. It is, perhaps, the most widely studied LCS subcategory, as it includes the study of FTLEs [40,58] within its scope. In this section, a brief background of geometric LCS theory is presented, followed by definitions of quantities required for extending the theory to sparse data. The section is concluded by an example demonstrating the influence of sparsity on geometric LCS analysis.

**Figure 1.**
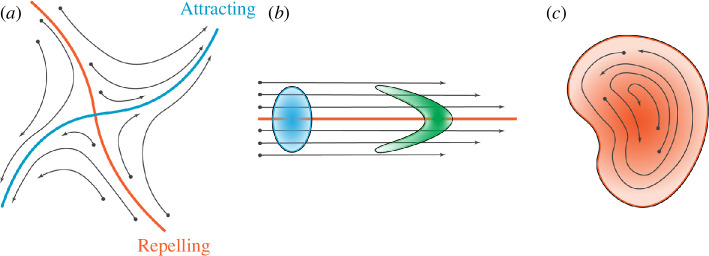
Categories of geometric LCS. (*a*) Hyperbolic LCS identify manifolds of attraction and repulsion. (*b*) Parabolic LCS are shear-driven and can be thought of as Lagrangian jet cores. (*c*) Elliptic LCS consist of regions of coherent rotation.

Geometric LCS theory is thoroughly discussed in the seminal review and textbook by Haller [12,15]. LCS are defined as codimension-1 manifolds (a two-dimensional (2D) line or a three-dimensional (3D) surface) that segment the flow into regions of quantitatively similar behaviour over a finite observation period. All geometric LCS can be classified into one of three categories: hyperbolic structures (defined as either attracting or repelling manifolds), parabolic structures (shear-driven manifolds thought of as Lagrangian jet cores) and elliptic structures (thought of as Lagrangian vortices). The three categories of structures can be seen in figure 1.

Elliptic LCS are often thought of as Lagrangian vortices defined over the time domain of interest. Therefore, in this work, elliptic LCS are defined, not as codimension-1 surfaces, but rather as material volumes containing everything inside the outermost ring that is defined by classical theory. The conceptualization of these structures in the literature is varied, as some authors choose to lump parabolic structures into the elliptic category [7]. In this sense, elliptic LCS are defined as regions of flow where neighbouring particles exhibit similar behaviour (rotational, in the sense of a Lagrangian vortex, or translational as in the case of a Lagrangian jet core) over the time span. In this work, however, parabolic and elliptic LCS remain distinct.

### 2.1. The flow map

The fundamental quantity in any geometric LCS analysis is the flow map, which is derived from the evolution of material elements within a flow. The position 𝐱(t) of any passive tracer in a dynamical system is governed by the velocity field 𝐯(𝐱(t),t) , where individual trajectories are solutions of


(2.1) $$\begin{array}{ccc} & \frac{d}{dt}𝐱(t)=𝐯(𝐱(t),t). & \end{array}$$

Let $D\subseteq {\mathbb{R}}^{d}$ be the domain of the system being studied, where d=2,3 in most cases of interest for fluid flows. It is often convenient to include the initial condition ${𝐱}_{0}$ and initial time $t_{0}$ as parameters in the particle trajectory $𝐱(t;t_{0},{𝐱}_{0})$ . The particle trajectory may be obtained by integrating through the velocity field according to


(2.2) $$\begin{array}{ccc} & 𝐱(t;t_{0},{𝐱}_{0})={𝐱}_{0}+\int_{t_{0}}^{t}𝐯(𝐱(\tau ;t_{0},{𝐱}_{0}),\tau )d\tau , & \end{array}$$

where $𝐱(t_{0};t_{0},{𝐱}_{0})={𝐱}_{0}$ and where $𝐱(t;t_{0},{𝐱}_{0})$ constitutes the mapping


(2.3) $$\begin{array}{ccc} & {𝐅}_{t_{0}}^{t}:D\to D:{𝐱}_{0}\mapsto {𝐅}_{t_{0}}^{t}({𝐱}_{0})=𝐱(t;t_{0},{𝐱}_{0}). & \end{array}$$

For the sake of simplicity, dependence on initial conditions will be omitted from the notation unless it will aid in clarity. Thus, $𝐱(t;t_{0},{𝐱}_{0})$ may be referred to as 𝐱(t) , or even as 𝐱 , unless more specificity is required.

The mapping ${𝐅}_{t_{0}}^{t}({𝐱}_{0})$ is referred to as the flow map, and it brings material from an arbitrary reference location in D to another spatial location in D [34,35]. The flow map is smooth in both space ( $C^{3}$ ) and time ( $C^{1}$ ) [40] and is a diffeomorphism with inverse


(2.4) $$\begin{array}{ccc} & {𝐱}_{0}=({𝐅}_{t_{0}}^{t}{)}^{-1}(𝐱(t))={𝐅}_{t}^{t_{0}}(𝐱(t)), & \end{array}$$

ensuring that no two reference points may occupy the same spatial point at a given time. Other useful properties of the flow map stem from the local existence and uniqueness of solutions for initial value problems [40]


(2.5a) $$\begin{array}{rl} F_{t_{0}}^{t_{0}}(x_{0}) & =x_{0}, \end{array}$$

(2.5b) $$\begin{array}{cccc} & {𝐅}_{t_{0}}^{t+s}({𝐱}_{0}) & ={𝐅}_{s}^{t+s}\circ {𝐅}_{t_{0}}^{s}({𝐱}_{0})={𝐅}_{t}^{t+s}\circ {𝐅}_{t_{0}}^{t}({𝐱}_{0}). & \end{array}$$

Thus, the flow map over the full-time domain $\left[t_{0},\;t\right]$ can be viewed as the composition of many intermediate flow maps.

### 2.2. The flow-map Jacobian

The flow-map Jacobian represents the deformation gradient of the flow map along a trajectory 𝐱 starting at material point ${𝐱}_{0}$ and time $t_{0}$ and mapping to a spatial position 𝐱(t) at time t , and is given by


(2.6) $$DF_{t_{0}}^{t}(x_{0})=\nabla_{x_{0}}F_{t_{0}}^{t}(x_{0}),\;DF_{ij}(t;t_{0},x_{0})=\frac{\partial x_{i}(t;t_{0},x_{0})}{\partial x_{0,j}},\;\forall i,j\in [1,...\;\;,d].$$

The flow-map Jacobian governs the deviation of tracers within an ϵ -neighbourhood of the particle ${𝐱}_{0}$ . Consider the perturbation ${𝐲}_{𝟎}={𝐱}_{0}+\Delta {𝐱}_{0}$ , when $\Delta {𝐱}_{0}$ is very small. The relative position of the deformed perturbation is defined by


(2.7) $$\Delta x=F_{t_{0}}^{t}(y_{0})-F_{t_{0}}^{t}(x_{0})=DF_{t_{0}}^{t}(x_{0})\Delta x_{0}+O({\left\|\Delta x_{0}\right\|}^{2})=DF_{t_{0}}^{t}(x_{0})\Delta x_{0},\;\text{ as }\Delta x_{0}\to 0,$$

where the right-hand expressions are the result of a Taylor series approximation and the norm is Euclidean.

For notational convenience, the argument of the tensors will be dropped unless it is ambiguous to do so. Hence, $D{𝐅}_{t_{0}}^{t}\equiv D{𝐅}_{t_{0}}^{t}({𝐱}_{0})$ , and so on. Throughout this work, other tensors will be introduced, and it will be assumed that the argument is ${𝐱}_{0}$ unless otherwise stated.

### 2.3. FTLEs

The right Cauchy−Green strain tensor is another fundamental element of the theory of geometric LCS and is defined as the Gram matrix of the flow-map Jacobian


(2.8) $$\begin{array}{ccc} & {𝐂}_{t_{0}}^{t}={\left(D{𝐅}_{t_{0}}^{t}\right)}^{⊤}D{𝐅}_{t_{0}}^{t}, & \end{array}$$

where $(\cdot {)}^{⊤}$ represents the matrix transpose; ${𝐂}_{t_{0}}^{t}$ is a Gramian matrix and, therefore, has the useful properties of being symmetric and positive semi-definite. Since the continuum assumption of fluids enforces one-to-one behaviour of the flow map, it is guaranteed that $\det\;D{𝐅}_{t_{0}}^{t}>0$ , and therefore that ${𝐂}_{t_{0}}^{t}$ is, in fact, positive definite and has d real eigenvalues.

Physically, the right Cauchy–Green strain tensor represents the squared change in local distances owing to the flow map ${𝐅}_{t_{0}}^{t}$ . Thus, the maximum magnitude of the perturbation at time t is determined by the $L^{2}$ norm on $D{𝐅}_{t_{0}}^{t}$


(2.9) $$\begin{array}{ccc} & \max\left\|\Delta 𝐱\right\|={\left\|D{𝐅}_{t_{0}}^{t}\right\|}_{2}\left\|\Delta {𝐱}_{0}\right\|=\sqrt{\lambda_{\max}({𝐂}_{t_{0}}^{t})}\left\|\Delta {𝐱}_{0}\right\|, & \end{array}$$

where $\lambda_{\max}$ represents the largest eigenvalue of ${𝐂}_{t_{0}}^{t}$ . Additionally, owing to the properties of the $L^{2}$ operator norm [59], ${\left\|D{𝐅}_{t_{0}}^{t}\right\|}_{2}$ may be computed as the largest singular value of $D{𝐅}_{t_{0}}^{t}$ and represents the maximal gain induced by the tensor.

The scalar value $\sqrt{\lambda_{\max}({𝐂}_{t_{0}}^{t})}$ may be viewed as an exponential, such that


(2.10) $$\begin{array}{ccc} & \sqrt{\lambda_{\max}({𝐂}_{t_{0}}^{t})}=e^{\sigma_{t_{0}}^{t}({𝐱}_{0})|\Delta t|}, & \end{array}$$

where $t_{0}+\Delta t=t$ . The exponent $\sigma_{t_{0}}^{t}$ is the FTLE, which represents the exponential growth rate of a linear deformation over the observation time. Because $\sigma_{t_{0}}^{t}$ assumes linearity, it can only be reliably evaluated for finite Δt if Δ𝐱 is infinitesimal, or for finite Δ𝐱 if Δt is infinitesimal. Therefore, it is typically computed according to


(2.11) $$\begin{array}{ccc} & \sigma_{t_{0}}^{t}=\lim_{\Delta {𝐱}_{0}\to 0}\;\frac{1}{|\Delta t|}\ln\;\sqrt{\lambda_{\max}({𝐂}_{t_{0}}^{t})}. & \end{array}$$

Objectivity is typically a concern when computing Lagrangian measures. A scalar quantity is said to be objective if it exhibits invariance under Euclidean transformations of the form


(2.12) $$\begin{array}{ccc} & \tilde{𝐱}=𝐐𝐱+𝐩, & \end{array}$$

where 𝐐 is a proper orthogonal rotation tensor and 𝐩 is a translation. Vector- and tensor-valued quantities are objective if they transform simply according to a proper orthogonal tensor (change of basis) [15,34].

### 2.4. Numerical approaches for computing Jacobians

**Figure 2.**
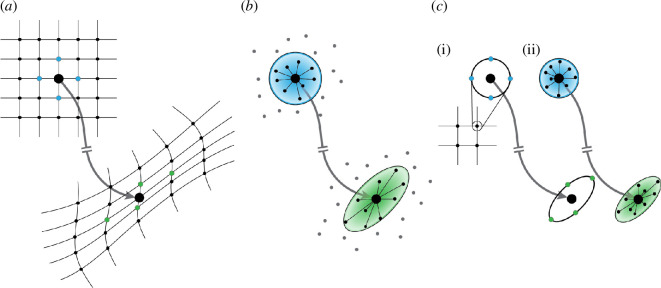
Diagrams of computation schemes for the 2D flow-map Jacobian. (*a*) Finite-differences approach. (*b*) Regression approach. (*c*) Planet-satellite approach, which can be accomplished using either (*i*) finite differences or (ii) regression.

Geometric LCS are typically computed using either finite differences or regression as the numerical engine. A short description of these methods is provided here along with figure 2. The finite-differences approach to computing $D{𝐅}_{t_{0}}^{t}$ was the first technique to be developed [10,40] and begins by artificially seeding massless particles throughout the flow domain in a fine mesh. Velocity information for the flow—in the form of an analytical expression or snapshots from simulation or experiment—is then used to propagate the positions of the particles over some finite-time domain $\left[t_{0},t\right]$ to their deformed positions using a numerical integrator. The gradient of the deformation (which is the flow-map Jacobian at the initial time) is then approximated at each particle with respect to the initial positions using a finite-differencing scheme. A schematic of this process is shown in figure 2*a*.

In some instances, the finite-differences algorithm for computing $D{𝐅}_{t_{0}}^{t}$ is inefficient. In circumstances where particle information is known on an unstructured mesh, for instance, superfluous steps are required to compute the LCS. To address this, Lekien & Ross [51] developed a method for computing $D{𝐅}_{t_{0}}^{t}$ using Voronoi cell-weighted least-squares regression on unstructured meshes [51]. This method was adapted to experimental particle flows by Raben *et al*. [53], where regression was used on the observed particle trajectories themselves to compute LCS quantities. The regression approach to the LCS approximation is shown as a schematic in figure 2*b*.

A third computational scheme was employed in this work to enable direct comparison of gradient estimation approaches on a pointwise basis. The method, referred to as the planet-satellite (PS) approach and illustrated in figure 2*c*, involves specifying an array of test locations—for example, on a uniform grid—then seeding numerical tracers around them to locally perform gradient computations at the test locations with either finite differences or regression. The primary advantage of this approach is that it allows multiple strategies to be evaluated at a specific spatial location and compared directly. As an example, one could vary the spacing between the centre particle and its satellites and evaluate the influence of the variation on gradient quality. This method will become especially useful in §4 when LGR is applied to finite-time gradient estimation.

### 2.5. Nonlinear effects dominate sparse data

Computing accurate geometric LCS is predicated on achieving very small $\Delta {𝐱}_{0}$ . For a finite-time domain, the validity of equation (2.7) breaks down as $\Delta {𝐱}_{0}$ increases. If an LCS analysis is still performed when $\Delta {𝐱}_{0}$ is large, the computed flow-map Jacobian does not accurately describe the tracer deformation as the tracers it is computed from are non-negligibly influenced by flow nonlinearities.

To explore this principle, a case study is presented. Consider the analytical flow field of the unsteady double gyre. The velocity field is defined by


(2.13a) $$\begin{array}{rl} u & =-\pi \;A\;\sin\;\left(\pi f(x,t)\right)\;\cos(\pi y), \end{array}$$

(2.13b) $$\begin{array}{rl} v & =\pi \;A\cos\;\left(\pi f(x,t)\right)\sin\;(\pi y)\frac{\partial f(x,t)}{\partial x}, \end{array}$$

where


$$\begin{array}{rlrlrl} f(x,t) & =a(t)x^{2}+b(t)x,\;\;\; & a(t) & =\epsilon \sin(\omega t),\;\;\; & b(t) & =1-2\epsilon \sin(\omega t), \end{array}$$

with parameters ϵ=0.1 , A=0.1 and ω=2π/10 . The computational domain over which trajectories are integrated is [0,2] in x by [0,1] in y with $t_{0}=0$ and t=15 , such that particles are advected through 1.5 periods of the flow.

**Figure 3.**
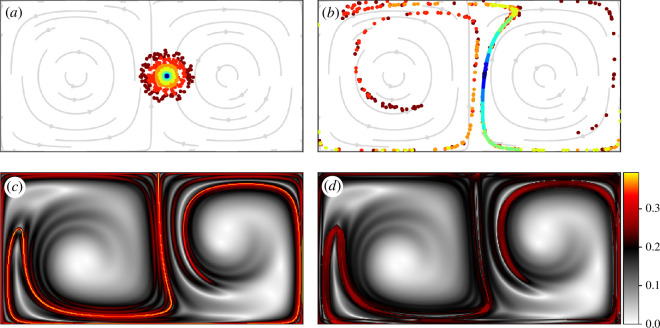
A demonstration of the nonlinear influence of the flow on the accuracy of the Jacobian for particles with large initial radius. (*a*) Particles are organized by initial radius. (*b*) Location of particles from (*a*) after 15 time units in the unsteady double gyre flow. (*c*) FTLE field evaluated at the level of grid spacing $\Delta {𝐩}_{0}$ ( $\Delta {𝐩}_{0}=\Delta {𝐱}_{0}=0.005$ : light blue particles). (*d*) FTLE field evaluated with $\Delta x_{0}=5\Delta {𝐩}_{0}$ ( $\Delta {𝐱}_{0}=0.025$ : green particles). All of the computations are performed using the PS approach with finite differences (figure 2*c,i*).

To demonstrate the influence of $\Delta {𝐱}_{0}$ on approximating $D{𝐅}_{t_{0}}^{t}$ , tracer neighbourhoods with varying radii are used to compute FTLE fields on the flow. Figure 3*a* shows a cloud of particles at time $t_{0}$ centred at ${𝐱}_{0}$ with varying radii indicated by colour. The particles are advected to the final positions (which are displayed in figure 3*b*), where it is clear that the deformation of particles with large initial radii (green to red particles) cannot be reasonably approximated by a linear transformation. However, as the initial radius decreases (blue particles), the deformation of the particles approximates an ellipsoid and can therefore be sufficiently described by a linear operator. It is important to note that, as the integration time increases, the radius that can be accurately approximated by the flow-map Jacobian decreases.

It is not clear simply by observing particle deformations how significantly the initial radius influences quantities used in identifying LCS. Figure 3*c,d* displays the FTLE field computed on the flow using different radii in the $D{𝐅}_{t_{0}}^{t}$ computations. In both cases, the PS method is used with finite differences (figure 2*c,i*), where the set of evaluation locations {𝐩} is a uniform grid and is kept the same between computations. In figure 3*c*, the FTLE computations are performed using satellite spacing $\Delta {𝐱}_{0}=0.005$ , which is equivalent to the grid spacing Δ𝐩 and the initial neighborhood radius of the light blue particles in figure 3*a,b*. Here, the FTLE field displays the sharp ridges that are consistent with the literature (see, for example, [13]). The computations in figure 3*d* use $\Delta {𝐱}_{0}=5\Delta 𝐩=0.025$ , which is the outer radius of the green particles in figure 3*a,b*, and the graphical representation uses the same colour mapping as for figure 3*c*. By visual inspection, it is seen that the regions from figure 3*c* with small FTLE values are largely unchanged, though the regions with large values (i.e. the FTLE ridges) are muted and distorted. Indeed, the dominant ridge that appears in the centre of the domain in figure 3*c* is difficult to identify in figure 3*d* as a result of the nonlinear warping effects of the flow. As one of the prevailing definitions of hyperbolic LCS is the ridges of the FTLE field [40], the field in figure 3*d* would identify spurious structures. Additionally, as $\Delta {𝐱}_{0}$ and Δt increase, the quality of the FTLE field decreases.

## 3. LGR

**Figure 4.**
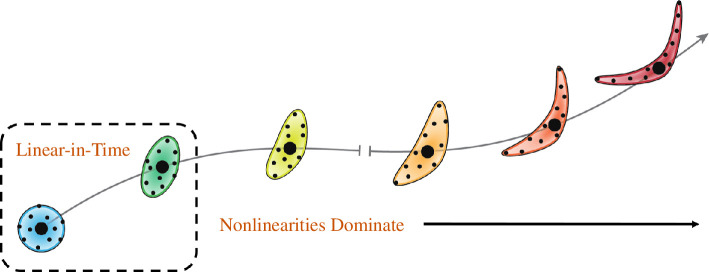
The deformation of a finite material neighbourhood can always be approximated by a linear operator when using a sufficiently short-time domain. Coloured ellipses represent the deformation of the blue circle along a trajectory indicated by the grey arrow. Black dots represent observable tracers in the flow.

In the previous example, it was demonstrated that the nonlinear deformations of material within a relatively large neighbourhood will obscure the structures identified in LCS procedures. However, if the deformations are considered over a sufficiently short duration, they can still be approximated by the flow-map Jacobian as a result of continuity. To visualize this, consider figure 4, which represents the deformation of a finite neighbourhood of material (on the left in a blue circle) over increasing durations. In the short time ( |Δt|/T≪1 , where T is the fastest timescale of the flow), the deformation of the material is approximately linear, transforming the circle into an ellipse. However, as the system evolves, the deformations become increasingly complex (yellow, orange and red blobs) and cannot be simply approximated by the flow-map Jacobian because the higher-order terms from equation (2.7) dominate.

In this section, the characteristics of short-time material deformations are considered and are presented as the foundation of extensions to computing hyperbolic and elliptic structures over arbitrary durations, which are discussed in subsequent sections.

### 3.1. Evolution equation of the flow-map Jacobian

Using the notation of equation (2.2), the temporal evolution of the flow-map Jacobian can be expressed as


(3.1) $$\begin{array}{ccc} & \frac{d}{dt}{D𝐅}_{t_{0}}^{t}({𝐱}_{0})=\frac{d}{dt}\left(\frac{\partial }{\partial {𝐱}_{0}}𝐱(t;t_{0},{𝐱}_{0})\right). & \end{array}$$

Exchanging derivatives and applying the chain rule yields


(3.2) $$\begin{array}{cccc} & \frac{d}{dt}D{𝐅}_{t_{0}}^{t} & =\nabla 𝐯(𝐱(t),t)D{𝐅}_{t_{0}}^{t}({𝐱}_{0}), & \end{array}$$

where $D{𝐅}_{t_{0}}^{t_{0}}({𝐱}_{0})={𝐈}_{d}$ , ∇𝐯(𝐱(t),t) is the spatial gradient of velocity at the particle position 𝐱 at time t , and ${𝐈}_{d}$ is the identity matrix in the space of the flow. Thus, the velocity gradient defines the evolution of the flow-map Jacobian [35,60]. When deformations are considered over an infinitesimal duration, they are therefore governed by the velocity gradient alone.

As it will become helpful in subsequent sections, the Cauchy–Stokes decomposition is introduced, which separates the velocity gradient into a rotational component and a dilatational component


(3.3) $$\begin{array}{ccc} & \nabla 𝐯=𝐖+𝐃. & \end{array}$$

The skew-symmetric spin tensor $𝐖=\frac{1}{2}(\nabla 𝐯-\nabla {𝐯}^{⊤})$ represents the rate of change of material rotation, and the symmetric stretch (or dilatation) tensor $𝐃=\frac{1}{2}(\nabla 𝐯+\nabla {𝐯}^{⊤})$ represents the rate of stretching in a material element. One application of these tensors is the computation of vorticity 𝛚 , which is defined as


(3.4) $$\begin{array}{ccc} & 𝐖𝐞=-\frac{1}{2}𝝎\times 𝐞,\;\forall 𝐞\in {\mathbb{R}}^{d}. & \end{array}$$

Moreover, the vorticity can be computed directly from the 2D velocity gradient by


(3.5) $$\begin{array}{ccc} & \omega_{z}=\frac{\partial v}{\partial x}-\frac{\partial u}{\partial y}=\nabla 𝐯[1,\;0]-\nabla 𝐯[0,\;1], & \end{array}$$

and in three dimensions by


(3.6) $$\omega_{3}={\left[\begin{array}{c} \frac{\partial w}{\partial y}-\frac{\partial v}{\partial z},\;\frac{\partial u}{\partial z}-\frac{\partial w}{\partial x},\;\frac{\partial v}{\partial x}-\frac{\partial u}{\partial y} \end{array}\right]}^{⊤}\;\;\;=\left[\begin{array}{c} \nabla v[2,\;1]-\nabla v[1,\;2]\\ \nabla v[0,\;3]-\nabla v[3,\;0]\\ \nabla v[1,\;0]-\nabla v[0,\;1] \end{array}\right],$$

where brackets indicate the zero-indexed m×n location in the velocity gradient tensor. In computations of vorticity performed throughout this work, equation (3.5) is employed.

Another velocity gradient-based quantity is the principal strain rate, which may be computed as the maximum eigenvalue of the strain rate tensor


(3.7) $$\begin{array}{ccc} & \epsilon_{1}=\lambda_{max}(𝐃), & \end{array}$$

and both 𝐖 and 𝐃 define the *Q*-criterion [36], given by


(3.8) $$\begin{array}{ccc} & Q=\frac{1}{2}\left({\left\|𝐖\right\|}_{F}^{2}-{\left\|𝐃\right\|}_{F}^{2}\right), & \end{array}$$

where ${\left\|\cdot \right\|}_{F}$ represents the Frobenius norm.

### 3.2. The velocity gradient in recent LCS literature

A variety of LCS analyses have been developed with explicit dependence upon the velocity gradient. Serra & Haller [61] use the spin and stretch tensors to construct variational formulae for computing objective Eulerian coherent structures, which were later implemented as a method for aiding search and rescue efforts [62]. Along these lines, the instantaneous Lyapunov exponent was recently developed using the first three Rivlin–Ericksen tensors to achieve a third-order accurate-in-time Taylor expansion of the right Cauchy–Green deformation tensor [63]. Additionally, the velocity gradient has been used in the development of the LAVD [43,60], which will be discussed in more depth in §5.

Implementing Lagrangian tools dependent on the velocity gradient remains a challenge owing to the difficulty of computing velocity gradients from trajectory data. A variety of methods for approximating gradients, divergence and vorticity have been developed in the oceanography community and have recently been surveyed [64,65]. In some instances, specific kinematic properties are computed directly. Divergence, for example, is often computed from tracers by the change in the area of polygons formed by tracers (e.g. Saucier [66]), and vorticity is estimated by evaluating the circulation around a polygon (e.g. Kawai [67]). Recent studies, however, have identified the linear least-squares approach of Molinari & Kirwan [68] to be a more effective means of estimating ocean velocity gradients [64,65,69]. This approach solves least-squares problems in the horizontal and vertical (*x* and *y*) directions using tracer velocities to approximate gradients along each axis. Recently, this approach has been used to approximate Lagrangian quantities [69].

While trajectory-based gradient estimation has been a part of the oceanography community for decades, it appears not yet to have made inroads into experimental fluid mechanics. With advances made in Lagrangian particle tracking (LPT) technologies [6,70], Lagrangian methods are gaining traction as the state-of-the-art in flow-field measurement. Gradient estimation in such studies is typically performed by interpolating on to a dense grid and performing numerical differentiation. Often, data-assimilation is implemented as post-processing to improve results [70]. As flow analysis domains grow larger, this process can be expensive both in computation time and in data storage demand. A fully Lagrangian approach to gradient estimation would reduce the costs associated with flow-field analysis in LPT studies.

This work proposes trajectory-based computation of flow gradients through the development of LGR. In this framework, deformation gradients are regressed from trajectories over short-times and then related to the velocity gradient. This tool was first presented by the authors Harms *et al*. [71] and is similar to the kinematic decomposition developed independently by Fenelon *et al*. [72] at the same time, which computes gradients at the centre of four-particle polygons formed by a Delaunay triangulation. The application of LGR to LCS quantities is discussed in §§4 and 5.

### 3.3. Estimating the velocity gradient using sparse trajectories

The connection between the flow-map Jacobian and the velocity gradient is expressed in equation (3.2). From it, the velocity gradient can be computed as a function of the flow-map Jacobian and its temporal derivative


(3.9) $$\begin{array}{ccc} & \nabla 𝐯=\frac{d}{dt}{D𝐅}_{t_{0}}^{t}{\left(D{𝐅}_{t_{0}}^{t}\right)}^{-1}. & \end{array}$$

When the magnitude of the time increment $\Delta t=|t-t_{0}|$ is much smaller than the fastest time-scales of the flow T such that |Δt|/T≪1 , the flow-map Jacobian and its inverse approximate the identity $D{𝐅}_{t_{0}}^{t}\approx D{𝐅}_{t}^{t_{0}}\approx {𝐈}_{d}$ . Additionally, the differential element of the flow-map Jacobian is approximated by its deviation from the identity $d\left(D{𝐅}_{t_{0}}^{t}\right)\approx D{𝐅}_{t_{0}}^{t}-{𝐈}_{d}$ . Incorporating these approximations into equation (3.9) approximates the velocity gradient from the flow-map Jacobian:


(3.10) $$\begin{array}{ccc} & \nabla 𝐯\approx \frac{D{𝐅}_{t_{0}}^{t}-{𝐈}_{d}}{\Delta t}, & \end{array}$$

where the result applies at time $t_{0}$ since the differential assumes that $t\to t_{0}$ . Therefore, an approximation of the velocity gradient is achievable using only trajectory information; it does not require any form of velocity information.

#### 3.3.1. Kernel-weighted least-squares regression of deformations

For equation (3.10) to be a valid approximation of the velocity gradient, it must first be possible to approximate $D{𝐅}_{t}^{\tau }$ from trajectory information. To accomplish this, the framework of kernel-weighted least-squares regression is used. This method differs from other regression-based approaches in the literature (see, for example, Lekien & Ross [51], Raben *et al*. [53] and Mowlavi *et al*. [7]) in that it is specifically applied over short-time intervals where deformations can be linearly approximated and that it specifies scale sensitivity using a kernel-weighting function.

The process of kernel-weighted least-squares regression of $D{𝐅}_{t}^{\tau }$ begins by identifying a particle of interest at time 𝐱(t) and selecting all of the surrounding particles ${𝐱}_{i}(t)$ within an ϵ -neighbourhood. These particles may be native to the flow or artificially seeded, depending on the application. The distance (typically Euclidean) from all neighbouring particles to the central particle at the initial time $\Delta {𝐱}_{i}(t)={𝐱}_{i}(t)-𝐱(t)$ is recorded in the matrix of differences at the initial time ${𝐗}_{t}\in {\mathbb{R}}^{d\times n}$ . These particles are deformed by the flow to their positions at time τ , and the new distances are recorded in a second matrix ${𝐗}_{\tau }\in {\mathbb{R}}^{d\times n}$ . The matrices ${𝐗}_{t}$ and ${𝐗}_{\tau }$ are constructed for n particles as


(3.11) $$\begin{array}{ccccc} & {𝐗}_{t}=\left[\begin{array}{cccc} | & | & & |\\ \Delta {𝐱}_{1}(t) & \Delta {𝐱}_{2}(t) & \cdots & \Delta {𝐱}_{n}(t)\\ | & | & & | \end{array}\right], & & {𝐗}_{\tau }=\left[\begin{array}{cccc} | & | & & |\\ \Delta {𝐱}_{1}(\tau ) & \Delta {𝐱}_{2}(\tau ) & \cdots & \Delta {𝐱}_{n}(\tau )\\ | & | & & | \end{array}\right], & \end{array}$$

such that $\Delta {𝐱}_{i}(\tau )={𝐅}_{t}^{\tau }({𝐱}_{i}(t))-{𝐅}_{t}^{\tau }(𝐱(t))$ represents the distance from the i ^th^ neighbour particle to the centre particle at time τ .

The deformed positions ${𝐗}_{\tau }$ can be viewed as a linear mapping from the initial positions ${𝐗}_{t}$ such that


(3.12) $$\begin{array}{ccc} & {𝐗}_{\tau }=𝐀{𝐗}_{t}. & \end{array}$$

This representation resembles the formulation of dynamic mode decomposition [73], as it fits a linear operator to describe the dynamics of material deformation from one instant in time to the next. The kernel-weighted least-squares regression problem identifies the optimal operator 𝐀 by solving the minimization problem


(3.13) $$\begin{array}{ccc} & 𝐀=\underset{𝐀}{\mathrm{arg min}}\left(\frac{1}{2}{\left\|{𝐊}^{\frac{1}{2}}\left({𝐗}_{\tau }-{𝐀𝐗}_{t}\right)\right\|}_{F}^{2}+\frac{\gamma }{2}{\left\|𝐀\right\|}_{F}^{2}\right), & \end{array}$$

where the kernel matrix $𝐊\in {\mathbb{R}}^{n\times n}$ is a design parameter and γ is the strength of regularization. The solution of the optimization is provided by


(3.14) $$\begin{array}{ccc} & 𝐀={𝐗}_{\tau }𝐊{𝐗}_{t}^{⊤}{\left({𝐗}_{t}𝐊{𝐗}_{t}^{⊤}+\gamma n{𝐈}_{d}\right)}^{-1}, & \end{array}$$

which is widely understood in the literature [74].

Because the operator 𝐀 maps particle positions at time t to their deformed positions at time τ , it represents an approximation of the flow-map Jacobian. Therefore,


(3.15) $$\begin{array}{ccc} & D{𝐅}_{t}^{\tau }\approx 𝐀. & \end{array}$$

Selecting a kernel matrix 𝐊 has a significant bearing on the quality of the operator identified by equation (3.14). Many construction strategies exist. Kernels are valid as long as 𝐊 is symmetric and positive semi-definite. Typically, one defines a kernel matrix according to a kernel function $k(\Delta 𝐱,\Delta {𝐱}^{\prime })$ , which defines the distance between data in the regression.

In this paper, **K** is either set to be the identity matrix, which ascribes equal weight to particles any distance from the centre particle, or particles are given weights according to a Gaussian function over the radius. In the latter case, the kernel function is defined


(3.16) $$\begin{array}{r} k_{RG}(\Delta x_{i}(t),\Delta x_{j}(t))=\left\{ \begin{array}{ll} \alpha^{2}\exp\left(-\frac{{\left(\Delta x_{i}(t)\right)}^{2}}{2l^{2}}\right), & \text{if}\;i=j\\ 0, & \text{otherwise} \end{array}\right., \end{array}$$

where the output scaling $\alpha^{2}$ and the input variance $l^{2}$ are hyperparameters. In practice, allowing $𝐊={𝐈}_{n}$ is the preferred approach when the particle spacing is already dense relative to the spatial scales of the flow or when the uncertainty of particle trajectories is larger. By equally weighting over the entire radial domain, the influence of spurious particles is reduced.

#### 3.3.2. Spatial scale sensitivity

Understanding which spatial scales of the flow contribute to gradient approximation is important when working with sparse or natural data. By examining the relative positions of tracers to the analysed trajectory, LGR provides a mechanism for approximating the scale sensitivity of the computed gradients. Consider, for example, two tracers embedded in a flow separated by some initial distance $r(t)={\left\|\Delta 𝐱(t)\right\|}_{2}$ . The approximated gradients are defined by the relative motion of the tracers to one another, which can only be observed at a length scale proportional to the initial separation of the two tracers. Using the Nyquist–Shannon sampling theorem, flow movements existing at scales less than 2r(t) will not be accurately sensed, and the lower-bound of physical scales that can be sensed by LGR is $2r_{min}(t)$ . In other words, at least two tracers need to exist in a flow feature (e.g. a vortex) for it to be sensed by LGR. Movements at scales smaller than this will not be sensed and may contribute to measurement error.

Determining the mean contributing spatial scale of the tracer cloud to the regressed gradient is also possible with the present description. This is most easily illustrated when the kernel-weighting matrix $𝐊={𝐈}_{d}$ , as the mean contributing scale, is calculated as the mean distance to neighbouring tracers


(3.17) $$r\;avg(t)=\frac{1}{n}\sum_{i=1}^{n}r_{i}(t),$$

where $r_{i}\left(t\right)=\;∥\Delta x_{i}\left(t\right){∥}_{2}$ )

In light of this, the kernel matrix 𝐊 may be viewed as a filter for flow scales sensed in the LGR operation, where weights can be tuned to highlight specific scales of motion in the flow. The effective scale contribution of kernel-weighted tracers can be calculated by considering the matrix of weighted tracer positions ${𝐘}_{t}={𝐗}_{t}𝐊$ . Each column $\Delta {𝐲}_{i}(t)\in {\mathbb{R}}^{d}$ in this new array represents the kernel-weighted relative position of the tracer ${𝐱}_{i}(t)$ . The proportional influence of each neighbouring particle on the gradient computation after weighting is given by


(3.18) $$\begin{array}{ccc} & p_{i}(t)=\frac{{\left\|{𝐲}_{i}(t)\right\|}_{2}}{\sum_{j=1}^{n}{\left\|{𝐲}_{j}(t)\right\|}_{2}}, & \end{array}$$

such that $0\leq p_{i}(t)\leq 1$ and $\sum_{j=1}^{n}p_{i}(t)=1$ . Multiplying this with the true physical distances $r_{i}(t)$ gives weighted distances, which can be averaged to compute the mean scale contribution under weighting


(3.19) $$\begin{array}{ccc} & r_{avg,\;weighted}(t)=\sum_{i=1}^{n}p_{i}(t)r_{i}(t). & \end{array}$$

As an example of the filtering effect of kernel weighting on effective scale sensitivity, the radial-Gaussian kernel of equation (3.16) will always reduce the mean contributing scale of LGR to estimated gradients, as it preferentially weights tracers near the central particle.

#### 3.3.3. The LGR algorithm

On sparse, randomly distributed tracer trajectories, LGR is implemented using the procedure outlined in algorithm 1. Typically, trajectory information is provided as a list of indexed tracers with position histories recorded. The algorithm first reorients the data to be indexed in time rather than by particle. Then, at each time step and for every particle, the nearest neighbours are determined and their relative positions at time $t_{i}$ and $t_{i+1}$ are recorded. Equation (3.14) is then used to compute the flow-map Jacobian and equation (3.10) is used to compute the velocity gradient. The result is stored with the tracer at time $t_{i}$ .

### 3.4. Results from the double gyre

**Figure 5.**
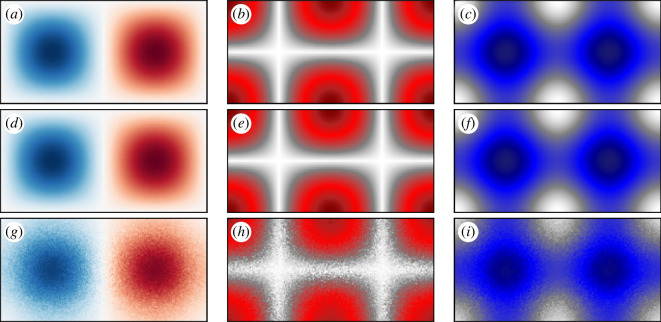
Comparison of quantities derived from the velocity gradient on the double gyre flow at t=0 using Lagrangian trajectories computed over Δt=0.01 . Vorticity is displayed in the first column, principal strain in the second and *Q*-criterion in the third. (*a,b,c*) Quantities computed analytically. (*d,e,f*) Quantities computed using equation (3.10) on a 200×100 uniform grid using Gaussian weighted regression on 30 random particles within radius r=0.01 of the evaluation point. (*g,h,i*) Same as (*d,e,f*) with r=0.5 .

To demonstrate the effectiveness of LGR for computing velocity gradients, velocity, principal strain and *Q* are computed both analytically and by LGR on the double gyre flow. All three are shown together to indicate that the entire velocity gradient is estimated by LGR. The results are displayed in figure 5.

To ensure that comparisons are accurate, both the analytical and LGR computations are performed on the same 200×100 uniform grid over the flow domain. Analytical values are computed directly from equation (2.13*b*) and are displayed in the top row of figure 5 where figure 5*a,b*, *c* represent the vorticity, principal strain and *Q,* respectively. LGR computations were performed using the PS approach where the centre particles were assigned to the grid points where analytical measurements were made. In the second row (figure 5*d,e,f*), results were computed using regression over 30 particles randomly placed with a uniform distribution within a radius of r=0.01 from the test particle. The kernel matrix 𝐊 was constructed using Gaussian weighting by radius (equation (3.16)) with no regularizer. In the final row (figure 5*g,h*,*i*), LGR was used with the same parameters as in the second row but with a sampling radius of r=0.5 for. Thus, the bottom row represents a distribution of particles that is 50 times more sparse than in the second row.


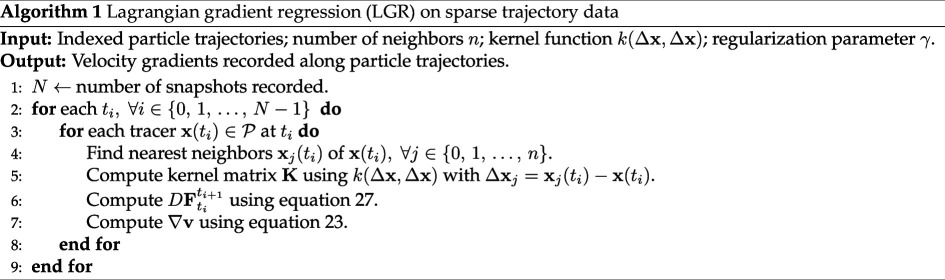


The results displayed in figure 5 demonstrate that LGR is a viable tool for computing complete velocity gradients (in n dimensions) directly from particle trajectories with a single regression. The fields computed using LGR over finely spaced particles (figure 5*d–f*) are indistinguishable from those computed analytically (figure 5*a–c*). The LGR results from sparse distributions (figure 5*g–i*) are noticeably noisier than the dense computations, which is a result of bias in the sampling distribution at the initial time.

The accuracy of this method, as with other trajectory-based approximation methods from the oceanography community (e.g. [64,65]), is dependent on the characteristics of the particle distribution at the time of estimation. Of particular importance is the distance between particles and the geometry of sampled particles. Multiple particles must exist on a gradient feature for it to be sensed. Thus, the spacing of tracers acts as a low-pass filter on the velocity gradient [71]. Additionally, asymmetric distributions can diminish gradient quality (see figure 5*g–i*). Kernel weighting helps to overcome this by specifying the scales of sensitivity when implementing LGR.

## 4. Computing hyperbolic and parabolic LCS using LGR

As discussed in §2, hyperbolic and parabolic LCS are generally computed from the right Cauchy–Green strain tensor ${𝐂}_{t_{0}}^{t}$ , which is the gram matrix of the flow-map Jacobian over the observed time domain. Because this quantity requires the measurement of material deformation over an extended duration, the sampling density of tracers must be large to ensure that the influence of nonlinearities remains small. On the other hand, in §3, it was discussed that material deformation over short periods of time can be accurately approximated from sparse trajectories since the sampling time Δt→0 , preventing the nonlinearities from distorting the result. To extend the framework of LGR to finite observation times—and therefore to the identification of hyperbolic and parabolic LCS—the theory of flow-map composition must be considered.

### 4.1. Computing Jacobians using composition

The use of flow map compositions for computing $D{𝐅}_{t_{0}}^{t}$ was pioneered by Brunton & Rowley [52] and Luchtenberg *et al*. [75] and has been applied in other studies such as that conducted by Raben *et al*. [53]. The theory stems from the uniqueness and existence properties of the flow map (equation (2.5a))—particularly the process property


(4.1) $$\begin{array}{ccc} & {𝐅}_{t_{0}}^{t_{n}}({𝐱}_{0})={𝐅}_{t_{n-1}}^{t_{n}}\circ \cdots \circ {𝐅}_{t_{1}}^{t_{2}}\circ {𝐅}_{t_{0}}^{t_{1}}({𝐱}_{𝟎}), & \end{array}$$

which states that any flow map from time $t_{0}$ to $t_{n}$ can be defined as the composition of n intermediate flow maps, as long as there are no gaps in the measurement times. Applying the chain rule to equation (4.1) yields


(4.2) $$\begin{array}{r} \begin{array}{rl} DF_{t_{0}}^{t_{n}}(x_{0}) & =D\left(F_{t_{n-1}}^{t_{n}}\circ \cdots \circ F_{t_{1}}^{t_{2}}\circ F_{t_{0}}^{t_{1}}(x_{0})\right),\\ & =D\left(F_{t_{n-1}}^{t_{n}}\left(F_{t_{0}}^{t_{n-1}}(x_{0})\right)\right)D\left(F_{t_{n-2}}^{t_{n-1}}\left(F_{t_{0}}^{t_{n-2}}(x_{0})\right)\right)\ldots D\left(F_{t_{0}}^{t_{1}}(x_{0})\right),\\ & =DF_{t_{n-1}}^{t_{n}}\left(F_{t_{0}}^{t_{n-1}}(x_{0})\right)DF_{t_{n-2}}^{t_{n-1}}\left(F_{t_{0}}^{t_{n-2}}(x_{0})\right)\ldots DF_{t_{0}}^{t_{1}}\left(x_{0}\right). \end{array} \end{array}$$

Then, recalling from equation (2.3) that $𝐱(t_{i})={𝐅}_{t_{0}}^{t_{i}}({𝐱}_{0})$ , the composition operation can be succinctly stated as


(4.3) $$\begin{array}{ccc} & D{𝐅}_{t_{0}}^{t_{n}}({𝐱}_{0})=\prod_{i=0}^{n-1}D{𝐅}_{t_{i}}^{t_{i+1}}\left(𝐱(t_{i})\right). & \end{array}$$

It is helpful to make some remarks regarding the composition framework. First, it is important to notice that equation (4.3) applies to every tracer in the flow for all times along its trajectory regardless of the method used to compute $D{𝐅}_{t_{i}}^{t_{i+1}}$ . Additionally, the important temporal constraint is that computational time domains are consecutive. Any given interval may progress forward in time, backwards in time or not at all, but all intervals must be connected.

A second remark is that, while previous methods involving flow map composition achieved their respective goals by performing interpolation to a grid at each time step [52,53,75], it is not a requirement for using flow map composition. As long as calculations are performed consecutively along the particle trajectory, no interpolation is necessary to achieve accurate results.

Often, when using composition to compute flow-map Jacobians, the group of particles identified at the initial time $t_{0}$ are tracked through intervals to their final positions at time $t_{n}$ . Computing the flow-map Jacobians $D{𝐅}_{t_{i}}^{t_{i+1}}$ at each interval and synthesizing through equation (4.3) to achieve the Jacobian over the full domain $D{𝐅}_{t_{0}}^{t_{n}}$ provides exactly the same results as if $D{𝐅}_{t_{0}}^{t_{n}}$ were computed using the initial and final times alone. Thus, composition alone does not solve the problem of sparse identification of LCS. To overcome this barrier, this work proposes the addition of resampling at each time step.

### 4.2. Composition with resampling

**Figure 6.**
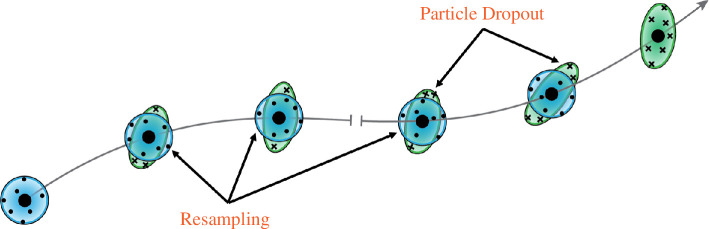
Schematic of the resampling procedure. At each time step, particles used in regression are resampled to ensure that each regression stays locally linear in time.


The process of composition with resampling is presented as a schematic in figure 6, which should be considered in comparison with the gradient computation process without resampling from figure 4. In the resampling paradigm, tracers that have exceeded a threshold radius from the analysed trajectory are discarded at each time step and replaced by others that are within closer proximity. As discussed in the previous section, if the time increment is small enough ( Δt/T≪1 ), the material deformation is approximately linear, and LGR can be used to fit the short-time Jacobian. Then, by applying equation (4.3), the complete Jacobian over time $\left[t_{0},\;t_{n}\right]$ can be accurately constructed.

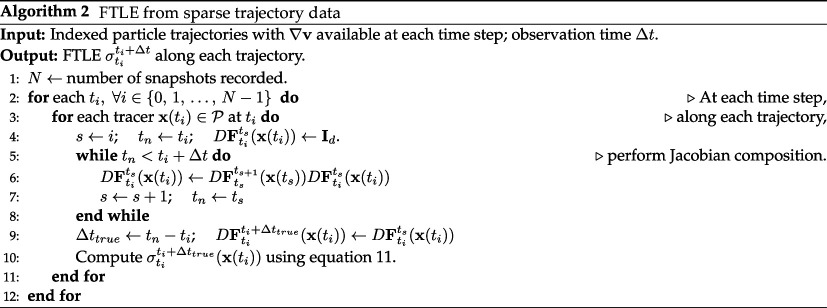


Depending on the context, resampling may be accomplished using artificial tracers or using those existing in the flow. If seeding using numerical tracers, one has control over the distribution of tracers in the regression neighbourhood. When computing directly from numerical tracers, some possible approaches include selecting all tracers within a specified radius or selecting the k -nearest neighbours to the analysed trajectory. In §6, when the tools developed in this work are demonstrated on sparse, random trajectories, the latter approach is performed. Regardless of how they are resampled, all tracers used in the regression must persist from one time step to the next to enable regression by equation (3.14). However, the neighbour particles may leave the domain outside the step that they are used in regression. The only particle that must persist the entire duration is the one for which the trajectory is analysed, which is a relaxation from other Jacobian computation methods since they require all particles to be visible for the entire duration.

### 4.3. Algorithmic implementation

Algorithm 2 may be used to implement composition for computing flow-1map Jacobians as described in this section. It is assumed that the velocity gradients or the intermediate flow-map Jacobians are already obtained along each particle trajectory. These may have been computed by LGR or they might be available through simulated velocity fields or analytical functions. Given these trajectories with stored intermediate Jacobians, equation (4.3) is applied to the stored Jacobians over some Δt at each time step to obtain the long-time flow-map Jacobian for each tracer. The FTLE along the trajectory is then computed using (equation 2.11).

### 4.4. FTLE performance comparison

**Figure 7.**
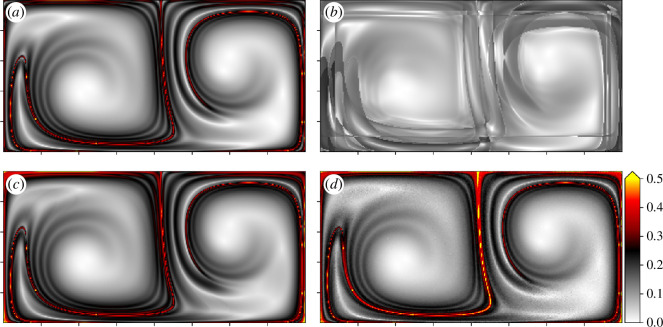
Performance comparison of FTLE computation schemes on the unsteady double gyre flow over 15 time units: (*a*) Baseline: FTLE computed using finite differences with no particle replacement for $\Delta x=10^{-6}$ by the PS method (figure 2 *c*,*i*). (*b*) Same as (*a*), but with large Δx=0.1 . (*c*) FTLE computed using finite differences (figure 2 *c*(i)) with particle replacement (figure 6), where initial spacing Δx=0.1 and replacement time Δt=0.1 . (*d*) Same as (*c*), but with regression (figure 2 *c*(ii)) using a Gaussian kernel for the weighting function.

The effectiveness of LGR with composition for hyperbolic and parabolic LCS detection is demonstrated by example on the double gyre flow. The parameters outlined with equation (2.13*b*) are used and Jacobians are computed over the time domain of t∈[0,15] . The idea of the experiment is to compute a baseline forward FTLE field on the flow using finite differences and small particle spacing and compare it to various results with large initial particle spacing. The result of the experiment is shown in figure 7 where the colour mapping function is kept the same in all frames.

The baseline FTLE field is displayed in figure 7*a* and is computed on a 400×200 grid of uniformly spaced interrogation points 𝐩 over the flow domain [0,2]×[0,1] . To ensure that the same interrogation points can be used between all methods, the PS computation scheme (figure 2*c*) is used. Neighbouring particles were placed with initial spacing $\Delta 𝐱=10^{-6}$ according to finite-differences sampling (figure 2*c*(i)), which is fine enough to ensure accurate results over the time domain but not small enough as to incur numerical precision error. For the computation of the Jacobian, central differences were computed using only the initial positions of the particle and their deformed positions at the final time t=15 . Note that the ridges are not always smooth and appear aliased in some areas. This is an artefact of the PS approach which occurs when Δ𝐩<2Δ𝐱 and is discussed further in §6.

Once again, only the initial and final positions were used to compute the field shown in figure 7*b*, which uses the same computational approach as figure 7*a,* differing only in the initial particle spacing. Here Δ𝐱=0.1 was used for satellite particle seeding around each interrogation point. Where initial particles were seeded outside of the flow domain either forward or backward differences were used, neglecting the particle seeded outside of the domain. The sharp features seen in the field are a numerical artefact that results from the smaller spacing of interrogation points compared to the spacing of satellite particles from which finite differences are computed Δ𝐩<Δ𝐱 . This is why, for example, the relatively fine feature in the lower left of the domain is still faintly visible even though the spacing of regression particles is larger than the feature. It should be noted that performing FTLE computations in this manner, while technically correct, is unconventional and has been used here solely for the purpose of direct comparison.

Figure 7*c,d* uses LGR with composition to achieve their results. In both instances, the initial spacing Δ𝐱=0.1 is the same as in figure 7*b*. Satellite particle replacement is employed every Δt=0.1 for a total of 150 compositions. Figure 7*c* uses structured sampling at each time step and finite differences (figure 2*c*(i)), whereas figure 7*d* samples 10 neighbouring particles uniformly at random within the radius r=0.1 and performs kernel regression (figure 2*c*(ii)) with the squared exponential kernel where $\alpha^{2}=1$ and $l^{2}=\text{var}(\|\Delta {𝐱}_{i}{\|}_{2})$ (the variance of radial distances of the satellites from the test particle). A regularizer is set at $\gamma =10^{-5}$ for numerical stability.

The results of this experiment demonstrate the effectiveness of the developed methods for accurately reconstructing the flow-map Jacobian from sparsely organized particles. Where the traditional computational strategy of figure 7*b* does not capture the fine details or the expected ridges seen in the baseline, both approaches using LGR with composition closely match the true values. The FTLE ridges that are typically used for identifying LCS are clearly present and identifiable, and all of the values in the field are commensurate with the baseline. In the control case in figure 7*b*, it is not evident that any FTLE ridges exist, and the higher values in the field are significantly diminished.

The errors in the fields generated using LGR with composition tend to be the highest near the boundaries of the domain, especially near the top boundary, towards which the particles rotate. This error may be attributable to issues with numerical sampling when the interrogation region is near the boundary. Effectively, the distribution of neighbouring particles is biased away from the location of the evaluation particle, skewing the results. In addition, it seems that the principal ridges exhibit slightly larger values than the baseline when using the kernel regression in figure 7*d* as opposed to finite-difference resampling in figure 7*c*.

## 5. Computing elliptic LCS from trajectories using LGR

Identifying elliptic LCS can be thought of as a Lagrangian approach to defining vortex boundaries. The first of such analyses was developed by Haller & Beron-Vera in 2012 [76] using a variational approach that identified closed bands of fluid that demonstrated minimal variability in their average straining. Later, Farazmand & Haller [77] defined polar LCS as closed and connected material surfaces (loops in two dimensions or tubes in three dimensions) of the PRA $\theta_{t_{0}}^{t}$ . LCS defined from the PRA, however, are only objective in two dimensions and can be difficult to interpret since $\theta_{t_{0}}^{t}\in [0,\;2\pi )$ . In response to this, Haller [60] derived the dynamic polar decomposition (DPD), which enabled rotationally coherent Lagrangian vortices to be identified by calculating the LAVD [43].

### 5.1. Background for elliptic metrics

Continuum mechanical analysis of rotation often involves polar decomposition, which states that $D{𝐅}_{t_{0}}^{t}$ has a unique decomposition


(5.1) $$\begin{array}{ccc} & D{𝐅}_{t_{0}}^{t}={𝐑}_{t_{0}}^{t}{𝐔}_{t_{0}}^{t}, & \end{array}$$

such that the rotation tensor ${𝐑}_{t_{0}}^{t}$ is proper orthogonal and the right stretch tensor ${𝐔}_{t_{0}}^{t}$ is symmetric, positive definite [34]; ${𝐑}_{t_{0}}^{t}$ represents a solid body rotation of a material element over the interval, and ${𝐔}_{t_{0}}^{t}$ represents its stretching.

The rotation tensor ${𝐑}_{t_{0}}^{t}$ admits a PRA $\theta_{t_{0}}^{t}$ , which has been used for the estimation of polar LCS [77]. As discussed by Haller *et al.* [43], however, ${𝐑}_{t_{0}}^{t}$ and $\theta_{t_{0}}^{t}$ are not dynamically consistent. That is, in general,


(5.2a) $$\begin{array}{rl} R_{t_{0}}^{t} & \neq R_{s}^{t}R_{t_{0}}^{s}, \end{array}$$

(5.2b) $$\begin{array}{cccc} & \theta_{t_{0}}^{t} & \neq \theta_{s}^{t}+\theta_{t_{0}}^{s}. & \end{array}$$

for two connected intervals $\left[t_{0},\;s\right]$ and [s,t] [60].

DPD was developed by Haller [60] to overcome these deficiencies. It splits any tensor defined by a linear process into a rotational process with zero rate of strain and an irrotational process with no vorticity. Specifically, the decomposition is defined as


(5.4) $$\begin{array}{ccc} & D{𝐅}_{t_{0}}^{t}={𝐎}_{t_{0}}^{t}{𝐌}_{t_{0}}^{t}, & \end{array}$$

where ${𝐎}_{t_{0}}^{t}$ is the proper orthogonal dynamic rotation tensor and ${𝐌}_{t_{0}}^{t}$ is the right dynamic stretch tensor. From this result, the dynamic rotation tensor ${𝐎}_{t_{0}}^{t}$ can be similarly decomposed into a relative rotation tensor ${𝚽}_{t_{0}}^{t}$ and a mean rotation tensor ${𝚯}_{t_{0}}^{t}$ such that


(5.4) $$\begin{array}{ccc} & D{𝐅}_{t_{0}}^{t}={𝚽}_{t_{0}}^{t}{𝚯}_{t_{0}}^{t}{𝐌}_{t_{0}}^{t}. & \end{array}$$

In Haller [60], it is shown that both ${𝐎}_{t_{0}}^{t}$ and ${𝚽}_{t_{0}}^{t}$ are dynamically consistent and ${𝚽}_{t_{0}}^{t}$ is objective.

The framework of DPD provides a robust basis for computing elliptic flow measures. The first metric considered is the intrinsic rotation angle (IRA):


(5.5) ψt0t(𝐱0)=12∫t0t|𝛚(𝐱(τ;t0,𝐱0),τ)−𝛚(τ)|dτ,

where 𝛚(t) represents the spatial average of vorticity. Physically, $\psi_{t_{0}}^{t}$ represents the angle swept by the relative rotation tensor over the interval. The LAVD is directly related to $\psi_{t_{0}}^{t}$ as


(5.6) $$\begin{array}{ccc} & {\text{LAVD}}_{t_{0}}^{t}=2\psi_{t_{0}}^{t}. & \end{array}$$

The dynamic rotation angle (DRA) $\varphi_{t_{0}}^{t}$ is defined as the angle generated by the dynamic rotation tensor ${𝐎}_{t_{0}}^{t}$ . It represents the amount of rotation that a fluid element experiences relative to the observer and can be computed using


(5.7) $$\begin{array}{ccc} & \varphi_{t_{0}}^{t}({𝐱}_{0};𝐠)=-\frac{1}{2}\int_{t_{0}}^{t}𝛚(𝐱(\tau ),\tau )\cdot 𝐠(𝐱(\tau ),\tau )d\tau , & \end{array}$$

where 𝐠 is an axis family related to the observer around which rotations are measured.

Both $\psi_{t_{0}}^{t}$ and $\varphi_{t_{0}}^{t}$ are dynamically consistent and can therefore be summed if computed on adjacent intervals. However, because of the dependence on the observer, $\varphi_{t_{0}}^{t}$ is not objective [60].

### 5.2. Computing LAVD and DRA from trajectories

Because LAVD and DRA depend on velocity gradients, they are typically not computed from sparse data (an exception is the work of Rypina *et al*. [69], which uses the method of Molinari & Kirwan [68] to estimate 2D velocity gradients from ocean drifters.) However, using LGR, such studies are possible because of the ability to calculate velocity gradients at each particle position along a trajectory.

Once velocity gradients have been computed according to the procedure defined in §3, the IRA $\psi_{t_{0}}^{t_{n}}$ and DRA $\varphi_{t_{0}}^{t_{n}}$ can be approximated by summing according to the relations


(5.8a) $$\begin{array}{rl} \psi_{t_{0}}^{t_{n}} & =\frac{1}{2}\sum_{i=1}^{n}\left|\omega (x_{i},t_{i})-\bar{\omega }(t_{i})\right|\Delta t_{i}, \end{array}$$

(5.8b) $$\begin{array}{cccc} & \varphi_{t_{0}}^{t_{n}} & =-\frac{1}{2}\sum_{i=1}^{n}𝛚({𝐱}_{i},t_{i})\cdot 𝐠(𝐱(t_{i}),t_{i})\Delta t_{i}, & \end{array}$$

where the spatially averaged vorticity


(5.9) 𝛚(ti)≈1N∑𝐱i∈Pω(𝐱i,ti)

is computed over all available particles P at time $t_{i}$ , and the axis family 𝐠 is typically regarded as unknown. It has been noted that, in especially sparse environments, 𝛚(ti) can be unreliable (see, for example, [14,57]), and thus should be used with care. In such cases, comparing with the DRA $\varphi_{t_{0}}^{t_{n}}$ may be useful even though it is not objective since it does not depend on spatial averages.

### 5.3. Demonstration on the double gyre

**Figure 8.**
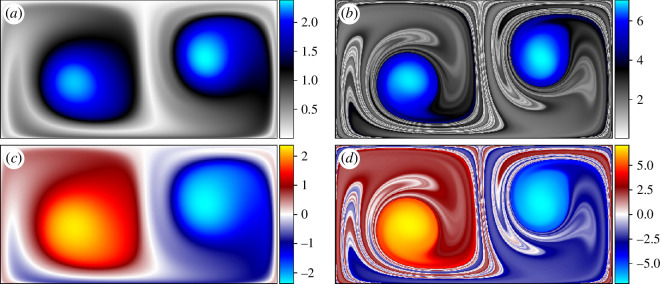
Examples of the Lagrangian metrics for elliptic LCS detection. Fields in the left column are computed on t∈[0,15] with Δt=0.1 on a uniform grid with $\Delta {𝐩}_{0}=0.005$ . Fields in the right column are the same except for t∈[0,45] . (*a,b*) IRA $\psi_{t_{0}}^{t}$ measured in complete rotations. (*c,d*) DRA $\varphi_{t_{0}}^{t}$ measured in complete rotations.

For context, the elliptic measures described in this section are presented on the double gyre flow. The IRA (half the LAVD) and the DRA are computed over t∈[0,15] in figure 8*a,c*, respectively, and again over t∈[0,45] in figure 8*b,d*. The relative Jacobians $D{𝐅}_{t_{i}}^{t_{i+1}}$ are with Δt=0.1 . Computations are performed along trajectories with initial positions uniformly spaced over the entire domain with $\Delta {𝐩}_{0}=0.005$ . The colour mapping is scaled to represent complete rotations of a fluid element, where a positive value represents a counterclockwise rotation in figure 8*c,d*.

Some observations should be made regarding figure 8. First, considering the IRA in figure 8*a*,*b*, the vortex cores (peaks in the $\psi_{t_{0}}^{t}$ field) are clearly visible as bright blue patches on either side of the domain. It is notable that the peaks are not centred with the peak in $|\omega_{z}|$ at any time in the computation, which is consistent with LCS and LAVD literature [43]. Additionally, the valleys of the $\psi_{t_{0}}^{t}$ field correspond to the ridges seen in the FTLE field.

Next, the DRA in figure 8*c,d* is considered. It should first be noted that the magnitude of rotations expressed in the colour bar is consistent with the magnitude of rotation seen in the intrinsic rotation fields. In both cases, the flow experiences just over 2.25 rotations in t∈[0,15] and close to 7 in t∈[0,45] . Here, it is also worthwhile to consider where the field is equal to zero. It is seen that these contours align with the ridges of the FTLE field in most (but not all) areas. Thus, the regions where average rotation is close to zero are highly correlated with the regions where there is the most stretching in a flow as indicated by the FTLE. However, since the DRA is not objective, the similarities between $\varphi_{t_{0}}^{t}$ and $\psi_{t_{0}}^{t}$ may be fewer in other, more complex flows.

## 6. Application of LGR to sparse data

LGR was introduced as a tool for identifying short-time flow-map Jacobians and velocity gradients in §3. It was then used to compute hyperbolic and parabolic LCS metrics in §4 and elliptic LCS metrics in §5. In this section, LGR is applied to fields of random particles with varied density and the quality of the results is evaluated.

The numerical experiments involve tracers of varying density propagated through the double gyre flow (equation (2.13*b*) with the associated parameters) on the spatial domain [0,2]×[0,1] from $t_{0}=0$ to t=15 . This allows for direct comparison to results presented earlier in the paper and ease of method evaluation. Neighbouring tracers are resampled every Δt=0.1 . Regression between snapshots is performed using k=15 nearest neighbours with the radial-Gaussian weighting function (equation (3.16)). The particle locations where values are computed are indicated in each figure as grey dots. Interpolation for the purpose of visualization occurs in between the points. When there are fewer than 1000 particles in the frame, radial basis function interpolation with a multiquadric kernel function is used to compute field values on a 200×100 uniform grid in x and y . Otherwise, a cubic scattered interpolation scheme is used.

The methods used in this work were designed to be effective on sparse data since it is expensive or infeasible to acquire dense measurement trajectories in many real-world applications. However, what constitutes a sparse distribution of data is contested. Aksamit *et al*. [57] suggest that sparsity should be determined by the number of tracers existing inside a unit volume of some characteristic length scale. This is an effective approach when the flow is characterized by a single dominant length scale but can be ambiguous and subjective if multiple scales are present. This approach may also be difficult to apply when tracers are not uniformly distributed throughout the flow. However, the double gyre does have a dominant length scale of approximately 1 unit, and so the notion of the sparsity of Aksamit *et al*. [57] may be quantified: the sparsity is, at minimum, 50 tracers per square unit and, at most, 1000 tracers per square unit.

Many authors who develop sparse LCS detection methods define sparsity relative to the requirements of traditional dense methods like FTLE (for examples, see [7,8,47], and the discussion of sparse methods contained in the review by Allshouse & Peacock [13]). This work assumes a similar definition of sparsity; since typical gradient-based LCS analyses on the double gyre often involve millions of tracers [13], the examples provided below are all sparse. This approach, too, can be subjective. It is for this reason that the discussion of the spatial sensitivity of the regression operation was provided in §3.3.2. While characterizing sparsity involves the subjectivity of either choosing a length scale or choosing a reference particle density, quantifying resolvable scales in an operation may be more objective.

### 6.1. Sparse computation of velocity gradients

**Figure 9.**
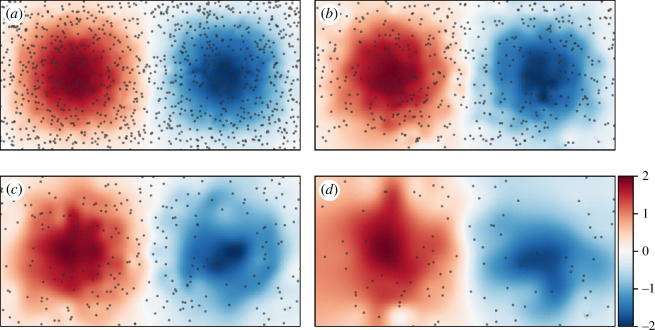
Vorticity computed on fields of sparsely distributed random particles using LGR. The velocity gradient is computed via algorithm 3.1, where $D{𝐅}_{t_{0}}^{t}$ is computed over t∈[0,0.1] using radial-Gaussian weighting on the k=15 nearest neighbour particles. Grey markers indicate the particle positions at $t_{0}=0$ . (*a*) 1000 particles. (*b*) 500 particles. (*c*) 250 particles. (*d*) 100 particles.

LGR is first demonstrated for its capacity to evaluate velocity gradients at an instance in time. Using randomly distributed particle trajectories, algorithm 1 is implemented to compute ∇𝐯 , from which vorticity $\omega_{z}$ is computed. For this demonstration, the regression time is over the domain $t_{0}=0$ to t=0.1 for all particle densities. The results are presented in figure 9, where figure 9*a* uses 1000 randomly distributed particles uses across the domain, figure 9*b* uses 500, figure 9*c* uses 250 and figure 9*d* uses 100. The colour mapping is chosen based on the analytically computed vorticity at $t_{0}=0$ in the double gyre flow and is the same as the scheme used in figure 5*a*,*d,g*. The grey markers represent the particle positions at the evaluation time $t_{0}=0$ .

Figure 9 demonstrates that vorticity—and more generally, the velocity gradient—can be accurately computed directly from Lagrangian trajectories recorded over short periods of time. In figure 9*a*, where the particle density is highest, the recorded vorticity matches very closely with the analytically computed field from figure 5*a*, which was computed on a uniform grid consisting of 20 times as many particles. In figure 9*b,c*, which contain 1/40 th and 1/80 th as many particles as figure 5*a*, respectively, the shape and magnitude of the vorticity field is consistent with the true values, though there are more errors owing largely to interpolation effects. Even in figure 9*d*, where only 100 particles are used, the centre of vorticity on either side of the vertical centre line is localized to the correct location and the magnitude of the vorticity does not deviate far from the true values.

The ability to compute velocity gradients directly from sparse trajectories is advantageous in the context of sparse measurements. Computing velocity gradients from tracer data can be difficult even when the recorded trajectories are abundant [78,79]. When the data are sparse, the difficulty is compounded. This challenge is typical when dealing with experimental flow-field measurements. For example, in figure 9*a*,*b*, the particle density is low, which limits the ability of PIV to achieve sufficient velocity field resolution for computing vorticity [4]. If PTV were used, the local particle velocity fields would need to be interpolated on to a grid prior to differentiation, which is expensive in terms of storage and computation time and can produce additional errors. The ability to accurately compute velocity gradients directly from sparse particles may help improve various analyses that depend on gradient-based quantities by reducing computational effort and storage requirements and improving accuracy. For a comparison of LGR with other velocity gradient computation pipelines common to experimental fluid mechanics, see Harms *et al*. [71].

### 6.2. Sparse computation of FTLE

In the next demonstration, FTLE fields are computed using algorithm 4.1. The results are compared with a baseline and with the approach of Mowlavi *et al*. [7]. The baseline approach computes Jacobians by regressing the deformation operator from the initial to the final states without resampling and using only connections between the examined particle and its neighbours in the regression. Mowlavi *et al*. expand on the baseline by storing all connections between particles in the regression matrices instead of only those with the centre particle. All three implementations identify the same k=15 nearest neighbours at time $t_{0}=0$ and perform analyses on the deformation up to t=15 . For the conventional approach and that of Mowlavi *et al*., the Jacobian is computed using unweighted ordinary least-squares regression ( $𝐊={𝐈}_{d}$ , γ=0 in equation (3.14)).

**Figure 10.**
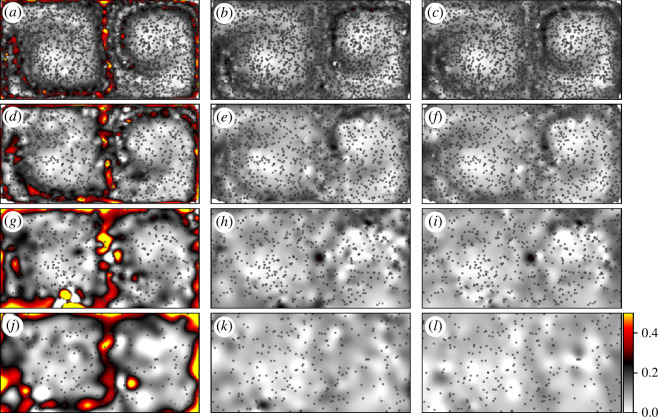
FTLE computed on fields of random particles using flow map composition with resampling (left column) using only the initial and final positions of particles in the conventional approach (centre column), and augmenting the data matrices according to Mowlavi *et al*. [7] (right column). Particles are advected in t∈[0,15] with resampling at Δt=0.1 . Jacobian computations use radial-Gaussian weighting on the k=15 nearest neighbour particles. Grey markers indicate the initial positions (evaluation locations) of the particles in the random field. (*a–c*) 2500 particles. (*d–f*) 1000 particles. (*g–i*) 500 particles. (*j–l*) 250 particles.

The results of the analysis are presented in figure 10, where figure 10*a*,*d*,*g*,*j* display the results using LGR, figure 10*b,e,h,k* displays the traditional results and figure 10*c*,*f,i,l* displays the results using the method of Mowlavi *et al*. Particle distributions are considered in decreasing density, with 2500 particles used in figure 10*a,b,c*, 1000 in figure 10*d,e,f*, 500 in figure 10*g,h,i* and 250 in figure 10*j*,*k,l*. In contrast, FTLE computations on this flow often use over $10^{6}$ particles [13]. The values indicated represent bounds on the true FTLE fields as seen in figure 7.

The performance improvement of LGR over other gradient approximation methods on sparse data is evident in figure 10. In all degrees of sparsity considered, FTLE via LGR displays evidence of the principal FTLE ridge down the centre of the domain. Moreover, the FTLE values that it identifies are commensurate with the values of the true field shown in figure 7. In contrast, only shadows of the ridges appear when other approaches are used and those only when the field is more densely seeded. In the sparsest cases considered (500 and 250 tracers), there is no evidence of dominant ridges in either the conventional method or the method of Mowlavi *et al*. It is thus concluded that LGR outperforms other available tools for sparse FTLE estimation and can be used as a tool for identifying hyperbolic LCS.

### 6.3. Sparse computation of elliptic metrics

**Figure 11:**
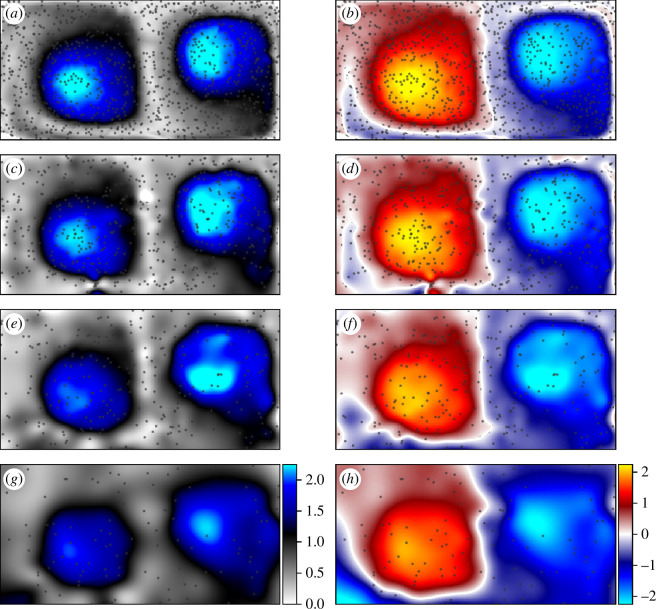
Lagrangian metrics for elliptic LCS identification computed from sparsely distributed fields of random particles. Quantities are computed over t∈[0,15] using regression of the k=15 nearest neighbour particles with radial-Gaussian weighting. Nearest neighbour particles used in the regressions are replaced every Δt=0.1 . Grey markers represent the particle positions at $t_{0}=0$ . First column: IRA $\psi_{0}^{15}$ . Second column: DRA $\varphi_{0}^{15}$ . (*a,b*) 1000 particles. (*c,d*) 500 particles. (*e,f*) 250 particles. (*g,h*) 100 particles.

The final demonstration computes elliptic LCS metrics from sparse trajectories as discussed in §5. The results are presented in figure 11. Specifically, the IRA $\psi_{t_{0}}^{t}$ and the DRA $\varphi_{t_{0}}^{t}$ are computed on the time domain t∈[0,15] . In the column on the left (figure 11*a,c,e,g*), results of $\psi_{t_{0}}^{t}$ are presented with increasing particle density, and in the right column (figure 11*b,d,f,h*), results of $\varphi_{t_{0}}^{t}$ are displayed for the same data as the left column. Computations are performed in figure 11*a*,*b* using 1000 randomly seeded trajectories, in figure 11*c,d* using 500, in figure 11*e,f* using 250 and in figure 11*g,h* using 100. The colour mapping represents the number of complete rotations observed by the measured quantity, and the maximum absolute value of the mapping is the same in both columns. Additionally, the mapped values can be compared to those of figure 8*a*,*c*, which demonstrate $\psi_{t_{0}}^{t}$ and $\varphi_{t_{0}}^{t}$ , respectively, on dense trajectories.

The results presented in figure 11 demonstrate that it is possible to accurately evaluate elliptic Lagrangian metrics from tracer data with no velocity information known *a priori*. Moreover, elliptic LCS features are seen to be robust to sparsity. In figure 11*a,b* where 1000 random particles are used in the computations, both the IRA $\psi_{t_{0}}^{t}$ and DRA $\varphi_{t_{0}}^{t}$ closely resemble their dense counterparts in figure 8. As the tracer density decreases, the form of the structures in the field remains consistent so that, even with only 100 particles seeded in the domain—as in figure 11*g,h*—the peaks and boundaries of the vortical regions of the flow are still readily observed.

Another feature of interest in figure 8 is the zero-contour of the DRA $\varphi_{t_{0}}^{t}$ . As discussed in §5, this contour represents the regions of the flow that experience net-zero rotation over the course of the flow. Because this contour lies between regions of opposite rotation, it will always exist in DRA fields where multiple vortices are present and will continuously divide them. Therefore, when dealing with sparse flows in practice, it may be convenient to use the zero-contour of the DRA as an approximation of hyperbolic LCS. From figure 11*b*,*d*,*f,h*, it is evident that this contour (seen as a white line in between coloured regions) is clearly identifiable even with few particles and that it approximately follows the FTLE ridges seen in figures 7 and 10. However, the contour does not exactly represent the FTLE ridges and is not objective. Therefore it should only be considered as an approximate LCS.

### 6.4. Structure sensitivity and robustness

When comparing the LCS results from sparse trajectories to those in previous sections where computations were performed on dense, structured data, it is apparent that the elliptic metrics and the velocity gradients are more robust to sparsity than the hyperbolic/parabolic LCS as revealed by the FTLE fields. In figure 10, for example, drawing precise ridges would be a challenging task to implement algorithmically for even a relatively dense field with 2000 random particles. On the other hand, the salient features of the elliptic LCS (figure 11) and velocity gradients (figure 9) are clearly visible with only 100 particles in the domain. This discrepancy results from the topology of the computed structures and their sensitivity to interpolation errors. Where elliptic LCS and velocity gradients are essentially measuring volumetric quantities, hyperbolic/parabolic LCS identify codimension-1 manifolds (material surface) of infinitesimal thickness that are more difficult to sense.

**Figure 12.**
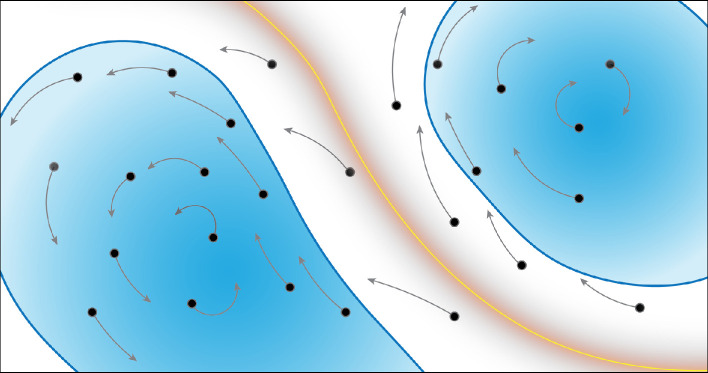
Illustration of geometric LCS emphasizing robustness to sparsity. The orange curve represents a hyperbolic/parabolic LCS, while the blue regions identify elliptic LCS. Because many tracers may exist on the elliptic LCS, they are more easily sensed in a flow than the manifolds of attraction, repulsion or shear.

To illustrate this further, consider the schematic in figure 12. Tracers and their trajectories are indicated alongside a repulsive hyperbolic LCS ridge (in orange) and two elliptic LCS (in blue), which hold tracers in Lagrangian vortices. Because the hyperbolic ridge is infinitesimally thin, it is exceedingly unlikely that any tracers exist immediately on top of the feature. Moreover, because the ridge is repulsive, the trajectories that begin near it diverge over time, allowing the nonlinear influence of the flow to further skew the results. The elliptic LCS, however, entrap particles within their boundaries for the observed duration. Since these particles exhibit similar rotational behaviour and are spread over finite volume, the feature is more likely to be sensed by identification algorithms. An implication of this is that elliptic LCS are more amenable to interpolation than hyperbolic LCS. Because a larger region of tracers is affected by elliptic LCS than hyperbolic LCS, it is much simpler to define a field from it over sparse data. These factors account for the improved robustness of elliptic LCS over hyperbolic and parabolic ones seen throughout this work and support the findings of other studies on LCS robustness such as that by Badza *et al*. [80].

## 7. Conclusions

This work presents methods for estimating velocity gradients and deformation gradients from sparse tracer trajectory data. The proposed methods are built upon the framework of LGR, which is introduced in §3 as an interpolation-free approach to computing velocity gradients from trajectories. Instead of using finite differences on a dense field of velocities, LGR computes gradients by regressing the linear operator governing the linear deformation of the tracer cloud over a short period of time. The velocity gradient can be computed directly from the regressed operator, or the product of many adjacent operators can be computed to estimate the total linear deformation (that is, the flow-map Jacobian) over finite intervals.

Estimating the gradient over short intervals effectively enables the use of sparse trajectories for gradient-based methods of detecting LCS. Where standard methods in LCS are built upon linearization in space, LGR is built on linearization in time. Therefore, by computing many intermediate gradients and synthesizing through composition, FTLE fields and other flow-map Jacobian-based metrics are reliably computed from sparse data with LGR. Moreover, since the short-time deformation is naturally related to the velocity gradient, methods like LAVD and DRA, which normally require dense fields of velocity gradients, can be computed along trajectories.

These methods were seen to perform well on sparse trajectory data from the analytical double-gyre flow model. In §6, LGR is shown to perform well at computing vorticity (and thus, velocity gradients), FTLE, LAVD and DRA data that are orders of magnitude sparser than the common approaches. Moreover, LGR is seen to show notable performance improvement in computing FTLE over other methods designed for sparse data.

Computing gradients using LGR has a variety of advantages:

1. Few approaches exist for computing velocity gradients using trajectory data, and they are mostly employed in the oceanographic setting. Applications in experimental fluid mechanics such as LPT could benefit from LGR studies by reduction of computational complexity and storage demand over standard gradient estimation algorithms. Computing gradients via LGR never require velocities to be computed or interpolation to be applied and, therefore, skips many steps in the typical LPT pipeline and avoids storing velocities and velocity gradients on a dense grid. It also enables a seamless transition to Lagrangian metrics since all gradient information is stored on the particle at each time step.
2. Most trajectory-based data can be sampled with a high resolution in time but not at a high resolution in space. Ocean drifters and LPT particles, for example, are sampled very quickly in time, but cannot typically be sampled densely enough in space for LCS analyses like FTLE. Because the sparsity-promoting aspect of LGR is the linearization of the flow in time rather than in space, it is naturally suited to realistic tracer data.
3. While the barriers identified by FTLE analysis are often used as diagnostics in LCS analyses, it is seen here that they are less robust to sparsity than elliptic measures. As autonomous applications are designed to operate with tracer data, it may be more convenient to seek structures through elliptic means than hyperbolic ones. Additionally, in situations where objectivity is not important, the DRA can be a useful metric for unsteady flow analysis.

Many avenues of future work exist for this project. First, as this work is designed to introduce LGR as a method, it has naturally been applied only to a simple and illustrative flow. As the tool develops, it should be applied to a broad range of flows of varying complexity and practicality. Additionally, work ought to be done to characterize the limits and potential of LGR. For example, it would be beneficial to explore other variations of the regression problem (for example, kernel regression) for their viability in learning from Lagrangian data. Ultimately, however, the goal of this research is to use Lagrangian tracer information in the decision-making protocol of autonomous technologies. Therefore, work is currently underway to assess the viability of the tools developed here for purposes of autonomy.

## Data Availability

We are committed to open science and reproducible research. To this end, we have made our code freely available for download so that researchers can easily apply our method to their own problems. Data and relevant code for this research work are stored in GitLab at https://code.stanford.edu/mckeon-group/LGR-for-the-detection-of-LCS-from-sparse-data and have been archived within the Zenodo repository: [81].

## References

[1] Lumpkin R , Centurioni L . 2019 NOAA global drifter program quality-controlled 6-hour interpolated data from ocean surface drifting buoys. In NOAA National Centers for Environmental Information.

[2] Manucharyan GE , Lopez-Acosta R , Wilhelmus MM . 2022 Spinning ice floes reveal intensification of mesoscale eddies in the western Arctic ocean. Sci. Rep. **12** , 7070. (10.1038/s41598-022-10712-z) 35488008 PMC9054753

[3] Hale RC , Seeley ME , La Guardia MJ , Mai L , Zeng EY . 2020 A global perspective on microplastics. J. Geophys. Res. **125** . (10.1029/2018JC014719)

[4] Raffel M , Willert CE , Scarano F , Kähler CJ , Wereley ST , Kompenhans J . 2018 Particle image velocimetry: a practical guide. Cham: Springer International Publishing.

[5] Nishino K , Liu Y , Adrian RJ . 1989 Measurement of lagrangian velocity by a particle tracking method. Center for turbulence research report. pp. 193–208. CTR-S89.

[6] Schanz D , Gesemann S , Schröder A . 2016 Shake-the-box: lagrangian particle tracking at high particle image densities. Exp. Fluids **57** , 70. (10.1007/s00348-016-2157-1)

[7] Mowlavi S , Serra M , Maiorino E , Mahadevan L . 2022 Detecting lagrangian coherent structures from sparse and noisy trajectory data. J. Fluid Mech. **948** , A4. 10.1017/jfm.2022.652. (10.1017/jfm.2022.652)

[8] Husic BE , Schlueter-Kuck KL , Dabiri JO . 2019 Simultaneous coherent structure coloring facilitates interpretable clustering of scientific data by amplifying dissimilarity. PLoS One **14** , e0212442. (10.1371/journal.pone.0212442) 30865644 PMC6415781

[9] Taira K *et al* . 2017 Modal analysis of fluid flows: an overview. AIAA J. **55** , 4013–4041. (10.2514/1.J056060)

[10] Haller G , Yuan G . 2000 Lagrangian coherent structures and mixing in two-dimensional turbulence. Physica D: Nonlinear Phenomena **147** , 352–370. (10.1016/S0167-2789(00)00142-1)

[11] Shadden SC . 2011 Lagrangian coherent structures. In Transport and mixing in laminar flows: from microfluidics to oceanic currents, pp. 59–89. Hoboken, NJ, USA: John Wiley & Sons, Ltd. (10.1002/9783527639748)

[12] Haller George . 2015 Lagrangian coherent structures. Annu. Rev. Fluid Mech. **47** , 137–162. (10.1146/annurev-fluid-010313-141322)

[13] Allshouse MR , Peacock T . 2015 Lagrangian based methods for coherent structure detection. Chaos **25** , 097617. (10.1063/1.4922968) 26428570

[14] Hadjighasem A , Farazmand M , Blazevski D , Froyland G , Haller G . 2017 A critical comparison of lagrangian methods for coherent structure detection. Chaos **27** , 053104. (10.1063/1.4982720) 28576102

[15] Haller G . 2023 Transport barriers and coherent structures in flow data: advective, diffusive, stochastic and active methods. Cambridge, United Kingdom: Cambridge University Press.

[16] Sapsis T , Haller G . 2009 Inertial particle dynamics in a hurricane. J. Atmos. Sci. **66** , 2481–2492. (10.1175/2009JAS2865.1)

[17] Shadden SC , Lekien F , Paduan JD , Chavez FP , Marsden JE . 2009 The correlation between surface drifters and coherent structures based on high-frequency radar data in monterey bay. Deep Sea Res. Part II Top. Stud. Oceanogr. **56** , 161–172. (10.1016/j.dsr2.2008.08.008)

[18] Reniers AJHM , MacMahan JH , Beron‐Vera FJ , Olascoaga MJ . 2010 Rip‐current pulses tied to lagrangian coherent structures. Geophys. Res. Lett. **37** . (10.1029/2009GL041443)

[19] Olascoaga MJ *et al* . 2013 Drifter motion in the gulf of Mexico constrained by altimetric lagrangian coherent structures. Geophys. Res. Lett. **40** , 6171–6175. (10.1002/2013GL058624)

[20] Filippi M , Rypina II , Hadjighasem A , Peacock T . 2021 An optimized-parameter spectral clustering approach to coherent structure detection in geophysical flows. Fluids **6** , 39. (10.3390/fluids6010039)

[21] Nolan PJ *et al* . 2018 Coordinated unmanned aircraft system (UAS) and ground-based weather measurements to predict lagrangian coherent structures (lcss). Sensors **18** , 4448. (10.3390/s18124448) 30558335 PMC6308849

[22] Green MA , Rowley CW , Haller G . 2007 Detection of lagrangian coherent structures in three-dimensional turbulence. J. Fluid Mech. **572** , 111–120. (10.1017/S0022112006003648)

[23] Mulleners K , Raffel M . 2012 The onset of dynamic stall revisited. Exp. Fluids **52** , 779–793. (10.1007/s00348-011-1118-y)

[24] Rockwood MP , Taira K , Green MA . 2017 Detecting vortex formation and shedding in cylinder wakes using lagrangian coherent structures. AIAA J. **55** , 15–23. (10.2514/1.J055051)

[25] Ahmed D , Javed A , Uz Zaman MS , Mahsud M , Hanifatu MN . 2023 Efficient sensor location for HVAC systems using lagrangian coherent structures. Math. Prob. Eng. **1** . (10.1155/2023/6059900)

[26] Amahjour N , García-Sánchez G , Agaoglou M , Mancho AM . 2023 Analysis of the spread of SARS-cov-2 in a hospital isolation room using CFD and lagrangian coherent structures. Phys. D: Nonlinear Phenom. 133825. (10.1016/j.physd.2023.133825) PMC10284441 37360502

[27] Yang K , Wu S , Zhang H , Ghista DN , Samuel OW , Wong KKL . 2021 Lagrangian-averaged vorticity deviation of spiraling blood flow in the heart during isovolumic contraction and ejection phases. Med. Biol. Eng. Comput. **59** , 1417–1430. (10.1007/s11517-021-02366-2) 34115272

[28] Nolan PJ , Foroutan H , Ross SD . 2020 Pollution transport patterns obtained through generalized lagrangian coherent structures. Atmosphere **11** , 168. (10.3390/atmos11020168)

[29] Tallapragada P , Ross SD , Schmale DG . 2011 Lagrangian coherent structures are associated with fluctuations in airborne microbial populations. Chaos **21** . (10.1063/1.3624930) 21974657

[30] Shadden SC , Taylor CA . 2008 Characterization of coherent structures in the cardiovascular system. Ann. Biomed. Eng. **36** , 1152–1162. (10.1007/s10439-008-9502-3) 18437573 PMC3886852

[31] Peng J , Dabiri JO . 2009 Transport of inertial particles by lagrangian coherent structures: application to predator–prey interaction in jellyfish feeding. J. Fluid Mech. **623** , 75–84. (10.1017/S0022112008005089)

[32] Shadden SC , Astorino M , Gerbeau JF . 2010 Computational analysis of an aortic valve jet with lagrangian coherent structures. Chaos **20** . (10.1063/1.3272780) 20370302

[33] Lekien F , Shadden SC , Marsden JE . 2007 Lagrangian coherent structures in n-dimensional systems. J. Math. Phys. **48** . (10.1063/1.2740025)

[34] Gurtin ME , Fried E , Anand L . 2010 The mechanics and thermodynamics of continua. Cambridge, United Kingdom: Cambridge University Press. (10.1017/CBO9780511762956)

[35] Truesdell C , Rajagopal KR . 2000 An introduction to the mechanics of fluids. Heidelberg, Germany: Springer Science & Business Media.

[36] Hunt JCR , Wray AA , Moin P . 1988 Eddies, streams, and convergence zones in turbulent flows. Center for Turbulence Research Report. pp. 193–208. CTR-S88.

[37] Jeong J , Hussain F . 1995 On the identification of a vortex. J. Fluid Mech. **285** , 69–94. (10.1017/S0022112095000462)

[38] Graftieaux L , Michard M , Grosjean N . 2001 Combining PIV, POD and vortex identification algorithms for the study of unsteady turbulent swirling flows. Meas. Sci. Technol. **12** , 1422–1429. (10.1088/0957-0233/12/9/307)

[39] Chakraborty P , Balachandar S , Adrian RJ . 2005 On the relationships between local vortex identification schemes. J. Fluid Mech. **535** , 189–214. (10.1017/S0022112005004726)

[40] Shadden SC , Lekien F , Marsden JE . 2005 Definition and properties of lagrangian coherent structures from finite-time lyapunov exponents in two-dimensional aperiodic flows. Phys. D Nonlinear Phenom. **212** , 271–304. (10.1016/j.physd.2005.10.007)

[41] Froyland G , Padberg K . 2009 Almost-invariant sets and invariant manifolds — connecting probabilistic and geometric descriptions of coherent structures in flows. Phys. D: Nonlinear Phenom. **238** , 1507–1523. (10.1016/j.physd.2009.03.002)

[42] Froyland G , Padberg-Gehle K . 2012 Finite-time entropy: a probabilistic approach for measuring nonlinear stretching. Phys. D: Nonlinear Phenom. **241** , 1612–1628. (10.1016/j.physd.2012.06.010)

[43] Haller G , Hadjighasem A , Farazmand M , Huhn F . 2016 Defining coherent vortices objectively from the vorticity. J. Fluid Mech. **795** , 136–173. (10.1017/jfm.2016.151)

[44] Froyland G , Padberg-Gehle K . 2014 Almost-invariant and finite-time coherent sets: directionality, duration, and diffusion. In Ergodic theory, open dynamics, and coherent structures, pp. 171–216. New York City, NY, USA: Springer.

[45] Hadjighasem A , Karrasch D , Teramoto H , Haller G . 2016 Spectral-clustering approach to lagrangian vortex detection. Phys. Rev. E **93** , 063107. (10.1103/PhysRevE.93.063107) 27415358

[46] Froyland G , Padberg-Gehle K . 2015 A rough-and-ready cluster-based approach for extracting finite-time coherent sets from sparse and incomplete trajectory data. Chaos **25** , 087406. (10.1063/1.4926372) 26328577

[47] Schlueter-Kuck KL , Dabiri JO . 2017 Coherent structure colouring: identification of coherent structures from sparse data using graph theory. J. Fluid Mech. **811** , 468–486. (10.1017/jfm.2016.755)

[48] Froyland G , Junge O . 2018 Robust FEM-based extraction of finite-time coherent sets using scattered, sparse, and incomplete trajectories. SIAM J. Appl. Dyn. Syst. **17** , 1891–1924. (10.1137/17M1129738)

[49] Schilling N , Froyland G , Junge O . 2020 Higher-order finite element approximation of the dynamic laplacian. ESAIM: Math. Model. Numer. Anal **54** , 1777–1795. (10.1051/m2an/2020027)

[50] Padberg-Gehle K , Schneide C . 2017 Network-based study of lagrangian transport and mixing. Nonlinear Process. Geophys. **24** , 661–671. (10.5194/npg-24-661-2017)

[51] Lekien F , Ross SD . 2010 The computation of finite-time lyapunov exponents on unstructured meshes and for non-euclidean manifolds. Chaos **20** , 017505. (10.1063/1.3278516) 20370295

[52] Brunton SL , Rowley CW . 2010 Fast computation of finite-time lyapunov exponent fields for unsteady flows. Chaos **20** , 017503. (10.1063/1.3270044) 20370293

[53] Raben SG , Ross SD , Vlachos PP . 2014 Computation of finite-time lyapunov exponents from time-resolved particle image velocimetry data. Exp. Fluids **55** , 1638. (10.1007/s00348-013-1638-8)

[54] Haller G , Aksamit N , Encinas-Bartos AP . 2021 Quasi-objective coherent structure diagnostics from single trajectories. Chaos **31** , 043131. (10.1063/5.0044151) 34251265

[55] Encinas-Bartos AP , Aksamit NO , Haller G . 2022 Quasi-objective eddy visualization from sparse drifter data. Chaos **32** . (10.1063/5.0099859) 36456328

[56] Kaszás B , Pedergnana T , Haller G . 2023 The objective deformation component of a velocity field. Eur. J. Mech. B. Fluids. **98** , 211–223. (10.1016/j.euromechflu.2022.12.007)

[57] Aksamit NO , Encinas-Bartos AP , Haller G , Rival DE . Relative fluid stretching and rotation for sparse trajectory observations. arXiv (10.48550/arXiv.2310.05500)

[58] Haller G . 2001 Distinguished material surfaces and coherent structures in three-dimensional fluid flows. Phys. D: Nonlinear Phenom. **149** , 248–277. (10.1016/S0167-2789(00)00199-8)

[59] Golub GH , Van Loan CF . 2013 Matrix computations. Baltimore, MD, USA: JHU press.

[60] Haller George . 2016 Dynamic rotation and stretch tensors from a dynamic polar decomposition. J. Mech. Phys. Solids **86** , 70–93. (10.1016/j.jmps.2015.10.002)

[61] Serra M , Haller G . 2016 Objective eulerian coherent structures. Chaos **26** , 053110. (10.1063/1.4951720) 27249950

[62] Serra M , Sathe P , Rypina I , Kirincich A , Ross SD , Lermusiaux P , Allen A , Peacock T , Haller G . 2020 Search and rescue at sea aided by hidden flow structures. Nat. Commun. **11** , 2525. (10.1038/s41467-020-16281-x) 32457536 PMC7250873

[63] Nolan PJ , Serra M , Ross SD . 2020 Finite-time lyapunov exponents in the instantaneous limit and material transport. Nonlinear Dyn. **100** , 3825–3852. (10.1007/s11071-020-05713-4)

[64] Essink S , Hormann V , Centurioni LR , Mahadevan A . 2022 On characterizing ocean kinematics from surface drifters. J. Atmos. Ocean. Technol. **39** , 1183–1198. (10.1175/JTECH-D-21-0068.1)

[65] Rypina II , Getscher TR , Pratt LJ , Mourre B . 2021 Observing and quantifying ocean flow properties using drifters with drogues at different depths. J. Phys. Oceanogr. **51** , 2463–82. (10.1175/JPO-D-20-0291.1)

[66] Saucier WJ . 1955 Principles of meteorological analysis. vol. 3. Chicago, IL, USA: University of Chicago Press.

[67] Kawai H . 1985 Scale dependence of divergence and vorticity of near-surface flows in the sea. J. Oceanogr. Soc. Jpn. **41** , 157–166. (10.1007/BF02111115)

[68] Molinari R , Kirwan AD . 1975 Calculations of differential kinematic properties from lagrangian observations in the Western Caribbean sea. J. Phys. Oceanogr. **5** , 483–491. (10.1175/1520-0485(1975)005<0483:CODKPF>2.0.CO;2)

[69] Rypina II , Getscher T , Pratt LJ , Ozgokmen T . 2022 Applying dynamical systems techniques to real ocean drifters. Nonlinear Process. Geophys. **29** , 345–361. (10.5194/npg-29-345-2022)

[70] Schröder A , Schanz D . 2023 3D lagrangian particle tracking in fluid mechanics. Annu. Rev. Fluid Mech. **55** , 511–540. (10.1146/annurev-fluid-031822-041721)

[71] Harms TD , Brunton SL , McKeon BJ . 2023 Direct computation of velocity gradients from particle trajectories. In 15th International Symposium on Particle Image Velocimetry, p. 1.

[72] Fenelon MR , Zhang Y , Schmid PJ , Cattafesta LN . 2023 Kinematic decomposition of multi-pulse volumetric particle tracking velocimetry data. In 15th International Symposium on Particle Image Velocimetry.

[73] Schmid PJ . 2010 Dynamic mode decomposition of numerical and experimental data. J. Fluid Mech. **656** , 5–28. (10.1017/S0022112010001217)

[74] Bishop CM , Nasrabadi NM . 2006 Pattern recognition and machine learning, p. 4. New York City, NY, USA: Springer.

[75] Luchtenburg DM , Brunton SL , Rowley CW . 2014 Long-time uncertainty propagation using generalized polynomial chaos and flow map composition. J. Comput. Phys. **274** , 783–802. (10.1016/j.jcp.2014.06.029)

[76] Haller G , Beron-Vera FJ . 2012 Geodesic theory of transport barriers in two-dimensional flows. Phys. D Nonlinear Phenom. **241** , 1680–1702. (10.1016/j.physd.2012.06.012)

[77] Farazmand M , Haller G . 2016 Polar rotation angle identifies elliptic islands in unsteady dynamical systems. Phys. D Nonlinear Phenom. **315** , 1–12. (10.1016/j.physd.2015.09.007)

[78] Etebari A , Vlachos PP . 2005 Improvements on the accuracy of derivative estimation from DPIV velocity measurements. Exp. Fluids **39** , 1040–1050. (10.1007/s00348-005-0037-1)

[79] Beresh SJ , Miller NE , Smith B . 2018 Practical challenges in the calculation of turbulent viscosity from PIV data. In 2018 Aerodynamic Measurement Technology and Ground Testing Conference, Atlanta, Georgia. vol. 2987. Reston, Virginia. (10.2514/6.2018-2987)

[80] Badza A , Mattner TW , Balasuriya S . 2023 How sensitive are lagrangian coherent structures to uncertainties in data? Phys. D Nonlinear Phenom. **444** , 133580. (10.1016/j.physd.2022.133580)

[81] TannerHarms . 2024 . TannerHarms/LGR-for-the-detection-of-LCS-from-sparse-data: Associated code for the RSOS article "Lagrangian Gradient Regression for the Detection of Coherent Structures from Sparse Trajectory Data" by Harms, Brunton, and McKeon (v1.0.0). Zenodo. (10.5281/zenodo.13126619) PMC11529625 39493296

